# A Novel Prairie Dog-Based Meta-Heuristic Optimization Algorithm for Improved Control, Better Transient Response, and Power Quality Enhancement of Hybrid Microgrids

**DOI:** 10.3390/s23135973

**Published:** 2023-06-27

**Authors:** Gagan Kumar Sahoo, Subhashree Choudhury, Rajkumar Singh Rathore, Mohit Bajaj

**Affiliations:** 1 Department of EE, Siksha ‘O’ Anusandhan (Deemed to be University), Bhubaneswar 751030, India; 2 Department of EEE, Siksha ‘O’ Anusandhan (Deemed to be University), Bhubaneswar 751030, India; 3 Cardiff School of Technologies, Cardiff Metropolitan University, Llandaff Campus, Western Avenue, Cardiff CF5 2YB, UK; 4 Department of Electrical Engineering, Graphic Era (Deemed to be University), Dehradun 248002, India; 5 Department of Electrical Engineering, Graphic Era Hill University, Dehradun 248002, India; 6 Applied Science Research Center, Applied Science Private University, Amman 11937, Jordan

**Keywords:** hybrid renewable energy sources, power quality, photovoltaics, fuel cell, battery, proportional integral (PI), bee colony optimization, thermal exchange optimization

## Abstract

The growing demand for electricity driven by population growth and industrialization is met by integrating hybrid renewable energy sources (HRESs) into the grid. HRES integration improves reliability, reduces losses, and addresses power quality issues for safe and effective microgrid (MG) operation, requiring efficient controllers. In this regard, this article proposes a prairie dog optimization (PDO) algorithm for the photovoltaic (PV)-, fuel cell (FC)-, and battery-based HRESs designed in MATLAB/Simulink architecture. The proposed PDO method optimally tunes the proportional integral (PI) controller gain parameters to achieve effective compensation of load demand and mitigation of PQ problems. The MG system has been applied to various intentional PQ issues such as swell, unbalanced load, oscillatory transient, and notch conditions to study the response of the proposed PDO controller. For evaluating the efficacy of the proposed PDO algorithm, the simulation results obtained are compared with those of earlier popular methodologies utilized in the current literature such as bee colony optimization (BCO), thermal exchange optimization, and PI techniques. A detailed analysis of the results found emphasizes the efficiency, robustness, and potential of the suggested PDO controller in significantly improving the overall system operation by minimizing the THD, improving the control of active and reactive power, enhancing the power factor, lowering the voltage deviation, and keeping the terminal voltage, DC-link voltage, grid voltage, and grid current almost constant in the event of PQ fault occurrence. As a result, the proposed PDO method paves the way for real-time employment in the MG system.

## 1. Introduction

### 1.1. General Background

In recent decades, electric grid networks have been involved in a variety of challenges, such as the use of fossil fuels and thermal production, which generates energy with crucial emissions that are costly, polluting, and fuel-depleted [1]. Subsequently, researchers have proposed the deployment of renewable energy sources (RESs) in the last few years because of the merits such as being environment friendly and abundantly available, energy security, decreased line losses, low operating costs, and ability to mitigate emissions and global warming [2]. Furthermore, due to the rising population and increased industry, the power demand has recently risen significantly. Hybrid renewable energy sources (HRESs) are being incorporated into the grid to meet the rising electricity demand, improve dependability, lower losses, and reduce the use of fossil fuels, which contribute to pollution [3]. Numerous HRESs such as photovoltaic (PV) [4], fuel cell [5], wind energy [6], and biomass [7] are integrated along with local loads and storage units to form a small entity of an electrical grid network termed a microgrid (MG) system [8]. In this article, the PV-based RES has been used for its numerous advantages such as [4] (1) no fuel cost, (2) extensive power expansion, (3) ease of operation even on a building’s rooftop, (4) low maintenance costs, (5) being plentily available, (6) being eco-friendly, (7) high reliability, and (8) being noiseless. To form an HRES, FC has been integrated with PV due to the following merits [5] (1) ease of use, (2) increased efficiency, (3) less hazardous pollutants, (4) increased modularity, (5) massive CHP, (6) increased stability, and (7) cost-effectiveness. But the major demerits associated with PV/FC systems are that PV supply is dependent on temperature and irradiance, FC response is sluggish, and at higher temperatures, the cost of the material rises, making it uneconomical to use. Therefore, this research article proposes the hybridization of battery-based energy storage units (due to high energy storage capability) [9] with PV/FC to competently meet the power demand as well as improve the performance, fuel economy, stability, and reliability of the MG system.

### 1.2. Motivation

The high penetration of HRESs into the MG network poses many challenges to stability and safe operation due to the intermittent nature of RESs, different types of sources (AC/DC) produced by RESs, several levels of generation of voltage by RESs, and the fact that they are environment-dependent [10]. The key to overcoming such difficulties is to employ power electronics interfaces (PEIs) that would maximize the power extracted from each HRES as well as convert the DC output to AC supply for necessary grid integration [11]. Nevertheless, the employment of PEIs in the MG system results in numerous power quality (PQ) issues, including the introduction of harmonics and system disruptions [11]. These disruptions must be addressed as they pose a threat to equipment on the load and source sides and may result in improper functioning and overheating. There are several types of PQ problems as reported in the literature such as harmonics, voltage sag, voltage swell, voltage interruption, oscillatory transient, flicker, notch, frequency deviation, etc. [12]. Of all of these problems, voltage interruption and voltage drop on the utility side cause the most problems, whereas harmonic distortion is on the customer side. To ensure the safe and consistent operation of the MG system, these PQ disturbances need to be substantially removed and efficiently dealt with. Due to their non-linear nature, these PEIs require robust control to increase power quality and efficaciously complete other control goals including voltage, frequency, and power flow management.

### 1.3. Literature Review

In this section, a comprehensive review of the available literature published by various researchers has been carried out regarding the different control strategies for optimal control of MG systems for enhanced power quality, efficient dynamic response, better energy management, and an overall increase in the efficiency of an MG system.

The use of classical controllers, such as proportional–integral (PI) controllers and proportional–integral–derivative (PID) controllers, is justified by their simplicity of installation, low cost, and quick computation times. However, the fact that PI and PID controllers are linear means they fail to respond robustly to these PQ disturbances. To obtain optimized control parameters of these linear controllers, many optimization methods have been applied. The authors of [13] have applied the Harris Hawks algorithm technique to optimize DVR controllers for improving the voltage quality of a low-voltage smart distribution system. The grasshopper optimization algorithm has been applied to D-FACTS for optimal tuning of PID control parameters to improve the power quality [14]. Masoud Dashtdar et al. have introduced a hybridization of the genetic algorithm and particle swarm optimization for enhancing the PQ of autonomous MG systems with voltage and frequency control [15]. The researchers in [16] have discussed the application of atom search optimization to bring about PQ enhancement in grid-connected PV/wind/battery with UPQC. Touqeer Ahmed Jumani et al. have applied the salp swarm optimization method to islanded MG for ensuring dynamic response and PQ improvement during faulted conditions [17]. A robust LFC-based African vulture optimization algorithm has been proposed by the authors for optimizing a hybrid MG’s frequency response under variable renewable source conditions [18]. Some of the other popular techniques used for PQ enhancement in MG are Aquila optimizer [19], fuzzy logic controller [20], adaptive-network fuzzy inference [21], green leaf-hopper flame optimization [22], SVM-based random subspace [23], chaotic butterfly optimization [24], deep convolutional neural network [25], artificial neural network [26], wavelet-fuzzy [27], support vector machine [28], model predictive control [29], stochastic dynamic programming [30], quantum teaching learning-based optimization [31], multi-agent system [32], crow search optimization technique [33], amended penguin optimization algorithm [34], and squirrel search algorithm [5]. Thorough study and analysis of popular contemporary algorithms available in the literature have been conducted. The merits and demerits have been summarized in Table 1 below for an easy understanding for readers and to evaluate the effectiveness of the new technique proposed in this research article.

A meticulous literature survey on all available popular optimization techniques (OTs) has been carried out and presented in Table 1. A critical analysis of the literature has led to the conclusion that there has been a push for improved versions of current algorithms or entirely new ones as there is no solitary OT that can deliver optimum results for all optimization issues. Additionally, there is also the requirement to deliver robust methods that will continually accomplish improved results. The result is that an OT’s resilience and efficacy are restricted to dealing with a certain set of challenges, rather than dealing with all possible problems. Numerous researchers have developed fresh, nature-inspired metaheuristic algorithms or enhanced tried-and-true ones, with varied degrees of success. In this regard, the authors of [35] have recently proposed a novel, nature-inspired method called prairie dog optimization (PDO) for solving unconstrained numerical optimization problems. This technique has not been explored much in the area of PQ enhancement in MG and has many advantages in comparison to other available techniques in the literature, which gave us the inspiration to apply it for the present study.

The following are the major merits of the PDO algorithm:

1. PDO is very capable of maintaining a well-balanced exploration and exploitation strategy.
2. Compared to the other algorithms, PDO has good efficiency and better abilities.
3. For real-world optimization issues with uncertain global optima, PDO is competent for predicting global optimum.
4. In comparison to other popular optimization techniques that have been studied, PDO exhibits more stable convergence.
5. Each clique performs optimization tasks within its domain or boundary, making effective use of the division of labour in the PDO.
6. The digging strength (DS) and predator impact (PE) qualities, which specifically affect the PDO updating process, are included in the models of the forage and burrow-building activities (exploration), communication, and anti-predation (exploitation) activities.

### 1.4. Major Contributions

The major contributions, motivation, and implications of this research article are described in brief below:

- Design, simulation, and optimal control of PV-, FC-, and battery-based HRESs for MG application with PQ enhancement in MATLAB/Simulink environment and application of suggested robust PDO algorithm to dynamically tune the PI gain parameters for ensuring improved PQ, system efficacy, and reliability.
- Verification of the efficiency and validation of the proposed controller by subjecting the HRES-based MG system to severe intentional PQ disturbances such as voltage swell, unbalanced load, oscillatory transient, and notch conditions. Furthermore, the evaluation of system characteristics and dynamics by comparing the proposed PDO technique with the traditional TEO algorithm, BCO algorithm, and PI controller.
- Comprehensive contrast study of different system characteristics at the grid side subjected to numerous PQ faults (swell, unbalanced load, oscillatory transient, and notch) such as active power, reactive power, apparent power, voltage deviation, power factor, frequency, THD, DC-link voltage, grid voltage, and grid current for the conventional and proposed techniques along with critical analysis of obtained numerical values for the suggested PDO technique and the conventional TEO, BCO, and PI methods by tabulating all control gain parameters (*K_p_* and *K_i_*) and system parameters (terminal voltage (Volt), DC-link voltage (volt), voltage deviation (p.u.), active power (watt), reactive power (Var), apparent power (VA), THD (%), power factor, frequency (Hz), grid voltage (p.u.), and grid current).

### 1.5. Paper Organization

**Figure 1:**
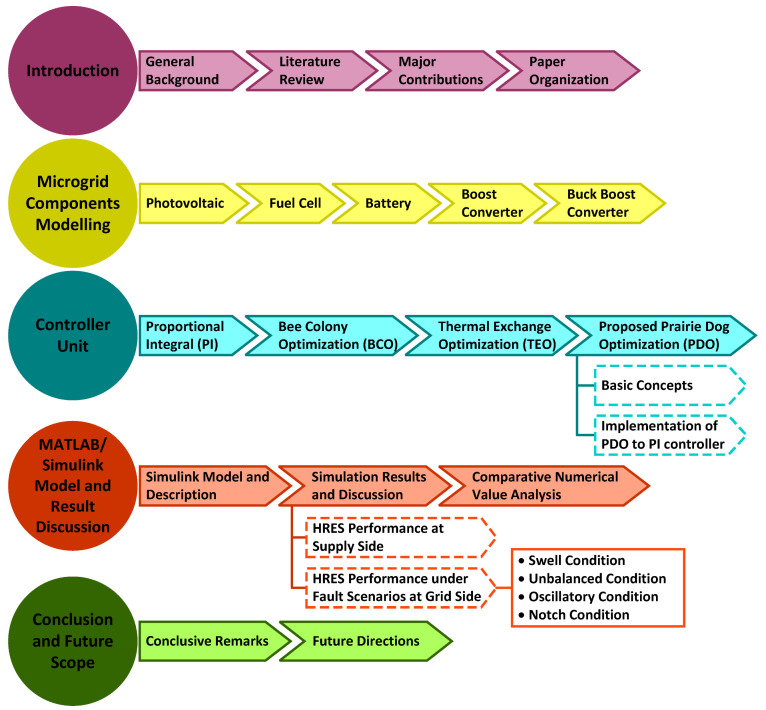
Graphical representation of the complete research study illustrating the primary ideas.

The complete research paper has been summarized as follows. Section 2 illustrates the detailed mathematical modelling of the HRES (PV/FC/battery)-based MG system. The proposed and traditional control approaches are discussed in detail in Section 3 with mathematical concepts and flowcharts. The MG system model designed in the Matlab/Simulink environment and the comparative results analysis for the conventional and proposed control methods are highlighted in Section 4. Finally, in Section 5, the conclusion is presented. Figure 1 is a diagrammatic illustration of the entire research work carried out highlighting the main concepts.

## 2. Microgrid Component Modelling

To justify the efficacy, reliability, and robust performance of the proposed PDO algorithm, a PV/FC/battery-based HRES for an MG network has been considered for this study and has been designed in Matlab/Simulink architecture. Furthermore, detailed modelling of all system components such as PV, FC, battery, and boost converter has been elaborated and the values considered for each power system unit have been provided in Appendix A.

### 2.1. Photovoltaic

A PV system converts solar energy into electrical energy using solar irradiance as a source of energy and is regarded as one of the most important renewable-based distributed energy resources because it is eco-friendly, has no noise pollution, requires minimal maintenance, and uses no fuel [36,37]. The basis of a PV system is the concept that solar cells convert light energy into electrical energy when it strikes them. The PV panel behaves as a current source, producing a photoelectric current that can be mathematically calculated from Equation (1) given below [4]:

(1) $$I=I_{L}-I_{0}\left[\exp\left(\frac{qV_{D}}{nKT}\right)-1\right]-\frac{V_{\;}}{R_{sh}}$$

### 2.2. Fuel Cell

A fuel cell (FC) is an electrochemical system that oxidizes fuel to generate electricity [5]. Researchers have reported several types of FCs in the literature. Of these, the proton exchange membrane fuel cell (PEMFC) is considered to be the most economical because of the following merits: (1) ease of use, (2) increased efficiency, (3) less hazardous pollutants, (4) increased modularity, (5) massive CHP, (6) increased stability, (7) cost-effectiveness, (8) flexibility in input fuel, (9) fast startup, (10) lightweight, (11) compact design, and (12) solidity of electrolyte [38]. An FC’s output voltage and its general equations can be represented as [5]:

(2) $$U=V-V_{1}-V_{2}-V_{3}$$

(3) $$V=N\left[V_{0}+\frac{RT}{2F}\ln\left(\frac{PH_{2}{\left(PO_{2}\right)}^{0.5}}{PH_{2}O}\right)\right]$$

(4) $$V_{2}=i\times R_{C}$$

(5) $$V_{1}=-A\times \ln\left(i\right)$$

(6) $$V_{3}=i\times R_{0}$$

### 2.3. Battery

Batteries are widely regarded as potent energy storage technology that can be employed in a wide range of electrical implementations. Due to their enormous energy storage capacity, they perform better than other available energy storage devices. Batteries are made up of components that convert electrical energy into chemical energy and vice versa [39]. Batteries provide a steady voltage and a significant lift. They can change the frequency through the absorption or injection of power into the load during frequency changes. Numerous types of battery-based energy storage units have been reported, like lead–acid, lithium-ion (Li-ion), nickel cadmium (Ni-Cd), nickel–metal hydride (Ni-MH), etc. However, Li-ion has normally been employed for application in MG systems due to many advantages such as (1) less self-discharging ability; (2) more charging and discharging cycles; (3) better efficiency; (4) higher energy density; and (5) being more economical [40].

Kirchhoff’s voltage law (KVL) is applied to a simplified battery model made up of a voltage source, resistances, and capacitors, and the output voltage of a Li-ion battery can be found as given in Equation (7) below [9]:

(7) $$V_{T}\left(t\right)=V_{OC}\left(z\left(t\right)\right)-V_{1}\left(t\right)-V_{2}\left(t\right)-R_{S}I\left(t\right)$$

### 2.4. Boost Converter

A boost converter is a DC-DC converter that enhances the output voltage concerning its supply voltage and functions through a pulse width modulation (PWM) technique for controlling the switching states of the switches [5]. It has a low to medium efficiency range. The output voltage is mathematically calculated as in Equation (8) [5]:

(8) $$V_{0\;}=\left(\frac{1}{1-D_{T}}\right)\times V_{S}$$

### 2.5. Buck/Boost Converter

The HRES employs a buck/boost converter with the objective of transmitting power in two directions. It has the characteristics of being lightweight and having compact volume and high reliability. Its ability to function as a buck converter and a boost converter in reverse directions depends on the functioning of two switches that are regulated by a PWM signal [41]. Both switches function simultaneously and alternately.

## 3. Controller Unit

### 3.1. Proportional Integral (PI) Controller

The PI controller is a conventional, linear, closed-loop, and easy-to-implement control method. It comprises the proportional and integral controller with gain parameters $K_{p}$ and $K_{i}$, respectively. $K_{p}$ enhances the rise time and $K_{i}$ reduces the steady-state error to ensure the overall control action. The primary drawback of the PI controller is that because of its linearity, it is unable to respond effectively to any non-linearities in the electrical grid network; as a result, additional techniques must be used to dynamically change the gain parameters. Mathematically, the PI controller can be presented as in Equation (9) [42]:

(9) $$X\left(t\right)=K_{p}\;e\left(t\right)+K_{i}\int e\left(t\right)\times dt$$

### 3.2. Bee Colony Optimization (BCO)

In the past, academics have been attentively examining the swarm behaviour of social insects to develop various intelligent optimization strategies. A unique kind of insect, honeybees can locate sustenance, i.e., nectar, even in erratic and dynamic environments, which makes them an enchanting insect [43]. Their effective foraging activity is mostly responsible for making this possible. Worker bees are the types of bees that are in charge of collecting and searching for nectar. They preserve the food they have collected for the bee community’s use in the future. The scout bee, a different type of bee, roams the region around its hive. Scout bees return to their hives after exploring new areas to notify the remaining bees of the location, nature, and abundance of available food sources. If these bees are successful in finding nectar, they perform a ritual dance known as the “waggle dance” inside the hive to entice and encourage their colony mates to follow them. A bee leaves its hive when it decides to follow the scout bee to gather nectar. It decides for one of the cases after returning [44]:

1. With a waggle dance before leaving the nectar spot, it can begin enlisting the help of its hive friends.
2. Without using any additional bees from the beehive, it can continue foraging at the discovered nectar supply.
3. It can become a loose follower and fully turn its back on the food source.

BCO mostly uses a hive of bees known as “artificial bees” that are looking for the best solution. Every artificial bee, unlike worker bees, is capable of coming up with a fresh idea. The forward and backward passes of each BCO iteration step are regarded as alternating phases.

(i) Forward Pass: Every artificial bee involved in this operation participates in the foraging process individually [45]. As a result, no information is shared during this stage. Specific motions are used to either generate a new component of the solution or modify an existing one as part of the exploration of new areas. As a function of the parameter FP, the number of moves carried out during a single forward pass is taken into account. It typically controls how quickly bees communicate information. In other words, it chooses how many passes forwards and backwards should be made throughout each cycle.

(ii) Backward Pass: All artificial bees share information about newly discovered places in the event of a backward pass. Keeping the best and worst options in mind, the information shared determines the quality of the partial solutions obtained.

Each bee decides whether or not it will remain dependable toward the nectar source depending on the quality of the solution. When artificial bees lose their loyalty, they become disloyal and are forced to join one of the recruiters’ suggested solutions [45]. The better ideas found by recruiters have a higher likelihood of being chosen for further investigation. A bee chooses whether to stick with the earlier discovered result after completing its forward pass. This decision is based simply on how good the result is compared to other results. Equation (10), given below, can be used to determine the likelihood that the *n*th bee will adhere to a previously discovered result [43]:

(10) $$p_{n}^{i+1}=e^{-\frac{s_{mn}-s_{n}}{i}}\;,\;\;n=1,2,\ldots ,B$$

where $s_{n}$ signifies the value of a partial or full solution normalized by the *n*th bee, $s_{mn}$ signifies the highest value of the overall partial/complete solution, and i reflects the number of forward passes i=1,2,…, FP).

By letting the uncommitted bees weigh the value of the solutions offered, a recruiter is chosen for all of the uncommitted bees. The mathematical formulation of the possibility that the *n*th partial/complete answer is chosen by any uncommitted bee is as follows [43]:

(11) $$p_{n}=\frac{s_{n}}{\sum_{a-1}^{R}s_{a}}\;,n=1,2,\ldots ,\;R$$

Here, the normalized value of the objective function for the *a*th promoted solution is indicated by $s_{a}$. R stands for the number of bees that are recruiters in this equation.

It can be used to solve a variety of challenging combinatorial optimization issues. In comparison to other techniques, it is more versatile and exhibits faster convergence with fewer configuration factors [43]. Nevertheless, the main demerits associated with BCO are as follows: (1) the study of the actions or characteristics of a single agent will not help to gather information about the whole swarm. Hence, choosing swarm-defeating behaviour will be difficult. (2) Since the action path is probabilistic, a single agent’s response is unpredictable and noisy, and (3) designing a swarm-based system is challenging since there is no analytical method.

### 3.3. Thermal Exchange Optimization (TEO)

According to the law of cooling by Newton, a body loses heat at a rate that is proportional to the temperature differential between it and its surroundings. According to Newton’s own words, the following describes the law of cooling: “For the iron’s heated air to always be blown away due to the wind and chilly air to alternately succeed it, and to obtain a heat level equivalent to the iron’s heat, the iron wasn’t set up in a quiet environment, nonetheless, in a wind that blew evenly onto it”. Several textbooks provide the contemporary lumped parameter technique for transient cooling. The physical characteristics are taken to be constant, and the heat transfer coefficient is taken to be h. The solid’s shape section is unimportant (except that h’s calculation will be impacted). When the object is suddenly relocated to a different environment, it is swiftly chilled to a consistent temperature by the fluid in the area. The object begins at a high temperature at time t=0. V is the solid’s volume, while A is its surface area.

The rate at which heat is lost through the surface can be mathematically denoted as [46].

(12) $$\frac{dQ}{dt}=h\left(T_{0}-T_{b}\right)A$$

where A= heat flow area; $T_{0}=$ high temperature; $T_{b}=$ constant temperature; h= temperature coefficient; Q= heat loss from the surface.

Heat loss over time dt is the same as the difference in heat as the temperature stored drops dT $h\left(T_{a}-T_{b}\right)A\;dt$ [46].

(13) $$V\;\rho cdT=-hA\left(T_{0}-T_{b}\right)dt$$

where V= volume; ρ= density; and c= specific heat.

The integration results in [47]

(14) $$\frac{T-T_{b}}{T_{0}-T_{b}}=\exp\left(-\frac{hA}{V\;\rho c}t\right)$$

The integration is only effectively valid when $\frac{hA}{V\;\rho c}$ does not change, i.e., not influenced by T, so it can be written as [47]

(15) $$\beta =\frac{hA}{V\;\rho c}$$

Equation (12) can be represented as [47]

(16) $$\frac{T-T_{b}}{T_{0}-T_{b}}=\exp\left(-\beta t\right)$$

Finally, Equation (12) may be rewritten as follows [47]:

(17) $$T=T_{b}+\left(T_{0}-T_{b}\right)\exp\left(-\beta t\right)$$

The opposite strategy from the TEO method has been adopted, where a few agents are designated as cooling items and the rest are meant to represent the environment [48]. TEO has many advantages such as [49] (1) minimum initial cost and operating cost; (2) low heat transfer area; (3) fewer pressure drops; and (4) capability of reducing the thermal resistance and obtaining a relatively high surface temperature. However, a few drawbacks associated with the TEO algorithm are that (1) the optimization problem is complicated and (2) the algorithm only allows for a certain number of iterations.

### 3.4. Proposed Prairie Dog Optimization (PDO)

#### 3.4.1. Basic Concepts

The PDO algorithm mimics the activities of four prairie dogs (PDs) to achieve optimization. The optimization problem domain is explored using the PDs’ feeding and burrow-building behaviours. The PDs’ tunnels are built upon a bountiful food source. As the current food source runs out, they search the entire colony or problem space for other food sources or solutions [35]. They create new tunnels around each new food source they uncover. The distinctive responses of the PDs have been employed for two different alarm or communication sounds. The sounds or signals used by PDs can indicate anything from the presence of predators to the availability of food. The PDs’ exceptional communication skills enable them to fulfil their nutritional requirements and defend themselves against predators. When the PDO is implemented, these two separate behaviours induce the PDs to gather in a certain spot or a promising area, after which exploitation is carried out to discover better or nearly ideal solutions. The herbivorous burrowing rodents known as PDs are found in the Great Plains, the southwest desert grasslands of the United States, the regions surrounding the plains and plateaus of Canada, and Mexico [50]. The PD is related to the Sciuridae family of squirrels, which also includes ground squirrels and chipmunks.

##### Inspiration of PDO

The five species of PDs are black-tailed, Gunnison’s, white-tailed, Utah, and Mexican. The black-tailed type is the one that is most frequently observed at Badlands National Park [35,51]. A PD weighs 1–3 pounds and can grow to a maximum length of 14–17 inches. PDs have acquired some morphological adaptations, such as short, muscular arms and very large toenails, to thrive in their environment. These characteristics allow them to run up to 35 mph over short distances and build tunnels to escape predators and reach the safety of their burrows [52]. PD habitats are exposed to natural threats like drought, prairie fires, floods, hailstorms, and blizzards.

##### Habitat and Burrowing

**Figure 2:**
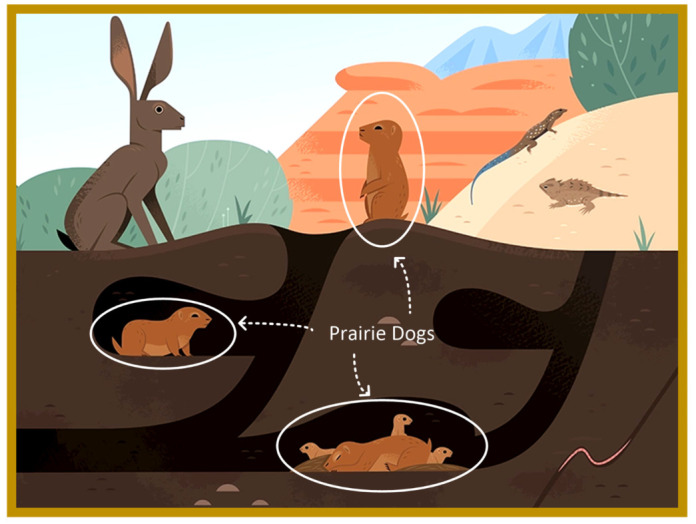
Burrow of prairie dogs [50].

The habitats are located between 2000 and 10,000 feet above sea level and have summer highs of 38 °C (100 °F) and winter lows of −37 °C (−35 °F). The PDs’ burrow homes give them vital defence against environmental hazards and aid in regulating their body temperatures [35,53]. A colony has 10 to 100 burrow openings per acre. Figure 2 shows the perimeter of the burrows. The ecosystem depends on the burrows. Changing the region’s soil composition lessens soil compaction and minimizes erosion by channelling rainwater into the water table.

The burrows are between 5 and 10 m long and 2 and 3 m deep, and the tunnel entrances (up to six) are typically 10 to 30 cm in diameter. The entrances to PDs’ burrows are typically just flat holes in the earth, but on rare occasions, PDs will build mounds of dirt to enclose them. The rim craters are 1 m high and the dome craters range in complexity and size from 20 to 30 cm [50]. Both craters protect the tunnel from flooding and aeration while also acting as observation posts for watching out for predators. In addition, the burrows have a variety of units for various uses, such as nursery chambers for their younglings, listening stations, chambers for night and winter, storerooms, and rear entrances for numerous escape routes, as depicted in Figure 2 [54].

##### Social Organization

The incredibly amiable PDs have underground colonies or communities. They construct their colonies underground to perfectly accommodate their natural surroundings. A PD colony with an area of 25,000 square miles has been reported. It is known that PDs live in large colonies (1–1000 acres) [50]. Regardless of the size of the colony, the subunits’ complications and capabilities are the same. Each family unit resides in a ward, which can hold around 10 to 30 family coteries, inside the colony or town. To keep the groupings stable, the female offspring usually remain in their natal coterie throughout their entire lives. The male children, however, typically leave their coterie once they reach sexual maturity in pursuit of a new family. PDs are fiercely territorial, and their lines of demarcation correspond to natural barriers like trees and rocks. A challenger from another family is repelled by the dominant male in a coterie to protect his territory [51].

PDs spend most of their days feeding, watching out for predators, digging new burrows, or maintaining those that already exist. Various components begin the optimization process of scavenging from one food source to another. Although they do consume some insects, PDs are primarily herbivorous. They travel from one place to another during the year, typically consuming grasses, very small seeds, and certain insects as they look for problems [53]. They lift their heads or stand on their hind legs while foraging to watch out for predators. The coterie looks for areas without existing burrows but that are well-suited for creating new burrows. A defined role that improves the town, ward, or coterie must be served by the location of the new burrows. In this way, exploring is enhanced much more.

##### Communication and Anti-Predation

One distinguishing attribute of PDs’ nature is communication, and the PDs’ unique response to different sounds makes it possible to use the recommended method. The majority of scientists concur that PDs possess a critical animal language that is yet to be discovered [35]. Although to us, the PDs’ “bark” might merely seem to be a simple squeak or yippee, to a PD, it has far deeper meanings [35]. The sounds or signals used by PDs can indicate anything from the presence of predators to the availability of food. The PDs’ ability to communicate has a substantial impact on their ability to ward off predators. They can distinguish between various predators and their distinctive hunting styles [55]. This behavioural adaptation is thought to result from the necessity for a varied reaction or set of survival abilities in response to the various predator hunting tactics. For example, if the signal identifies a hawk as the predator, only PDs in the hawk’s flight path hide, while others keep watching from their burrows [56]. In the case of coyotes, they only watch from their tunnel entrances, but when they see human predators, they collectively retreat into their burrows. Exploitation is accomplished using an exact reply to a particular sound. The coteries respond appropriately to the various distinct sounds, which are compared to calls to prospective places. The core of the suggested PDO is this ongoing cycle of behaviours [35].

#### 3.4.2. Mathematical Model Formulation and Algorithm of PDO

##### Assumptions and Implementation

To facilitate the creation of the models for the proposed PDO, the following presumptions were made [35], and the corresponding specification of parameters is provided in Appendix A:

1. Each prairie dog is a member of one of the m coteries that make up the colony, each of which has n prairie dogs.
2. The m groups of prairie dogs are further divided into identical subgroups.
3. Every coterie resides in a colony ward, or the problem search space equivalent.
4. Each ward has a minimum of ten burrow entrances, which increases to a hundred as nest building activities take place.
5. An antipredation call and a call for a new food supply (a new burrow being built) are two separate noises that are used.
6. Only individuals from the same coterie engage in foraging, burrow construction (exploration), communication, and anti-predation (exploitation) behaviours.
7. The exploration and exploitation actions are repeated m (number of coteries) times since other coteries in the colony are working on the same things at the same time.

##### Initialization

PDO uses an arbitrary initialization for the position of the PDs, just like other population-based methods. The populations of PDs serve as the search agents, and each PD is represented in d-dimensional space by a vector.

A coterie’s PDs are each a part of one of the n coteries. A vector may locate each PD inside a certain coterie because the PD exists and functions as a group or coterie. The matrix given below represents the position of each coterie (CT) in a colony [35]:

(18) $$CT=\left[\begin{array}{ccccc} CT_{1,1} & CT_{1,2} & \ldots & CT_{1,d-1} & CT_{1,d}\\ CT_{2,1} & CT_{2,2} & \ldots & CT_{2,d-1} & CT_{2,d}\\ \vdots & \vdots & CT_{i,j} & \vdots & \vdots \\ CT_{m,1} & CT_{m,2} & \ldots & CT_{m,d-1} & CT_{m,d} \end{array}\right]$$

where $CT_{i,j}$ stands for the ith coterie’s jth dimension. The equation below represents the position of each prairie dog in a coterie [35].

(19) $$PD=\left[\begin{array}{ccccc} PD_{1,1} & PD_{1,2} & \ldots & PD_{1,d-1} & PD_{1,d}\\ PD_{2,1} & PD_{2,2} & \ldots & PD_{2,d-1} & PD_{2,d}\\ \vdots & \vdots & PD_{i,j} & \vdots & \vdots \\ PD_{n,1} & PD_{n,2} & \ldots & PD_{n,d-1} & PD_{n,d} \end{array}\right]$$

where $PD_{i,j}$ stands for the ith prairie dog in a coterie’s jth dimension. According to the equations given below, a uniform distribution is used to distribute each CT and PD site [35].

(20) $$CT_{i,j}=U\left(0,1\right)*\left(UB_{j}-LB_{j}\right)+LB_{j}$$

(21) $$PD_{i,j}=U\left(0,\;1\right)*\left(ub_{j}-lb_{j}\right)+lb_{j}$$

where $ub_{j}=\frac{UB_{j}}{m}$, $lb_{j}=\frac{LB_{j}}{m}$, $U\;\left(0,\;1\right)$ is a uniformly distributed random number between 0 and 1. $UB_{j}$ and $LB_{j}$ are the upper and lower bounds of the jth dimension of the optimization problem respectively.

##### Fitness Function Evaluation

The defined fitness function receives the solution vector and computes the fitness function value for each PD’s location. The array shown below contains the values that are obtained [35].

(22) $$f\left(PD\right)=\left[\begin{array}{ccccc} f_{1}([PD_{1,1} & PD_{1,2} & \ldots & PD_{1,d-1} & PD_{1,d}])\\ f_{2}([PD_{2,1} & PD_{2,2} & \ldots & PD_{2,d-1} & PD_{2,d}])\\ \vdots & \vdots & \ldots & \vdots & \vdots \\ f_{n}([PD_{n,1} & PD_{n,2} & \ldots & PD_{n,d-1} & PD_{n,d}]) \end{array}\right]$$

The fitness function values for each PD represent the quality of the food available at a specific source, the ability to create new burrows, and the capability of responding effectively to anti-predation alerts. The achieved minimal fitness value is regarded as the best solution to the given minimization problem so far. The fitness function values are stored in an array that is sorted. For making burrows that aid in their ability to escape predators, the next three are taken into account along with the best value.

##### Exploration

**Figure 3:**
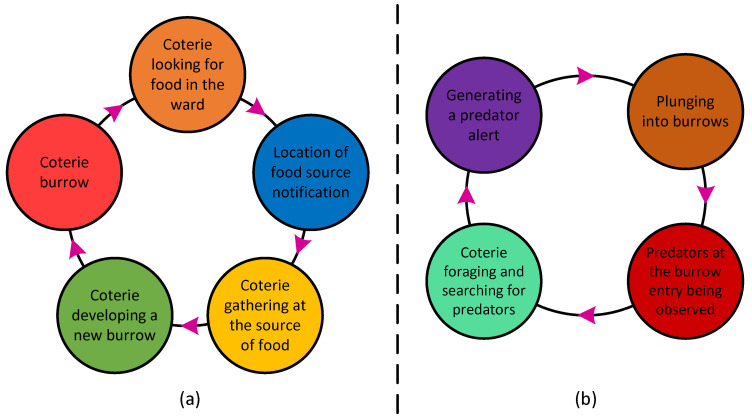
(**a**) Exploration strategy. (**b**) Exploitation strategy.

In this part, the PDO exploratory method is explained, as shown in Figure 3a. The optimization problem domain is explored using the PDs’ feeding and burrow building behaviours. The PDs’ tunnels are built upon a bountiful food source. As the current food source runs out, they search the entire colony or problem space for other food sources or solutions [35]. They create new tunnels around each new food source they uncover. The burrows are necessary for both environmental and predator defence. All PDs live in towns or colonies, and each colony is organized into family groups or coteries with specific colonial borders. Only the presence of a predator stops the several coteries from feeding and excavating burrows together within their boundaries.

Based on four criteria, PDO might choose between exploration and exploitation. The maximum number of iterations is divided into four parts: the first two are for exploration and the last two are for exploitation.

The two techniques for exploration are dependent on [35]

$$iter<\frac{Max_{iter}}{4}\;\mathrm{and}\;\frac{Max_{iter}}{4}\;\leq iter<\frac{Max_{iter}}{2}$$

whilst the two strategies for exploitation are dependent on

$$\frac{Max_{iter}}{2}\leq iter\leq 3\frac{Max_{iter}}{4}\;\mathrm{and}\;3\frac{Max_{iter}}{4}\leq iter\leq Max_{iter}$$

In the exploration phase, the coterie’s initial tactic is to have members scour the ward for fresh food sources. The Levy flight motion best depicts the motion of the PDs as they search for food. Because of the distinctive large hops, this movement prevents an exhaustive search of a given place yet effectively searches a variety of areas (exploration). They produce characteristic noises to alert other individuals of the discovery of food sources. Following the quality of the food source, the best is accessed, chosen for foraging, and new burrows are constructed. In the algorithm’s exploration phase, foraging position updating is provided by the equations given below [35].

(23) $$PD_{i+1,j+1}=GBest_{i,j}-eCBest_{i,j}\times \rho -CPD_{i,j}\times Levy\left(n\right)\;\forall \;iter<\frac{Max_{iter}}{4}$$

(24) $$PD_{i+1,j+1}=GBest_{i,j}\times rPD\times DS\times Levy\left(n\right)\;\forall \;\frac{Max_{iter}}{4}\leq iter<\frac{Max_{iter}}{2}$$

where $eCBest_{i,j}$ evaluates the effects of the most effective solution currently acquired worldwide and $GBest_{i,j}$ signifies the best solution currently available globally.

rPD signifies the location of a random solution, $CPD_{i,j}$ denotes the randomized cumulative effect of all prairie dogs in the colony, and ρ designates the experiment’s specialized food source alarm set at 0.1 kHz.

The calibre of the food source determines the coterie’s digging strength, indicated by DS, which has a random value. It is commonly known that the Levy distribution, Levy (n), encourages better and more efficient problem search space exploration [35].

(25) $$eCBest_{i,j}=GBest_{i,j}\times \Delta +\frac{PD_{i,j}\times mean\left(PD_{n,m}\right)}{GBest_{i,j}\times \left(UB_{j}-LB_{j}\right)+\Delta }$$

(26) $$CPD_{i,j}=\frac{GBest_{i,j}-rPD_{i,j}}{GBest_{i,j}+\Delta }$$

(27) $$DS=1.5\times r\times {\left(1-\frac{iter}{Max_{iter}}\right)}^{\left(2\frac{iter}{Max_{iter}}\right)}$$

where Δ stands for a modest number that signifies disparities that occur among the PDs and r introduces the stochastic property to confirm exploration and takes the value of −1 or 1 based on the present iteration. The maximum number of iterations is called $Max_{iter}$, while iter is the current iteration. The r adds the stochastic property to ensure exploration and takes the value of −1 or 1 depending on the current iteration.

##### Exploitation

This section comprehensively explains PDO’s exploitative practices. The entire phase is depicted using a schematic block diagram in Figure 3b. The proposed PDO takes advantage of the differential reactions of PDs to two separate alarms or communication noises. The sounds or signals used by PDs can indicate anything from the presence of predators to the availability of food [35]. The PDs’ exceptional communication skills enable them to fulfil their nutritional requirements and defend themselves against predators. Additionally, they can communicate details about the calibre of various food sources as well as information about predators and their hunting techniques. Each of them responds differently to these diverse noises; for instance, if the communication signals a reliable source of high-quality food, they assemble there to satiate their hunger (feed). Additionally, if the communication identifies a hawk as the predator, only PDs along the bird’s route go into hiding while the others remain watching from their burrows.

These two different behaviours cause the PDs to gather in one place or, in the case of PDO implementation, a promising location where additional search (exploitation) is carried out to find better or nearly ideal solutions. PDO’s exploitation procedures are designed to extensively scour the prospective regions found during the exploration phase.

The equation given below models the two tactics used for this phase. As was previously mentioned, PDO alternates between these two tactics under the conditions

$$\frac{Max_{iter}}{2}\leq iter\leq 3\frac{Max_{iter}}{4}\;\mathrm{and}\;3\frac{Max_{iter}}{4}\leq iter\leq Max_{iter,}$$

respectively [35].

(28) $$PD_{i+1,j+1}=GBest_{i,j}-eCBest_{i,j}\times \varepsilon -CPD_{i,j}\times rand\forall \;\frac{Max_{iter}}{2}\leq iter<3\frac{Max_{iter}}{4}$$

(29) $$PD_{i+1,j+1}=GBest_{i,j}\times PE\times rand\forall \;3\frac{Max_{iter}}{4}\leq iter<Max_{iter}$$

where $eCBest_{i,j}$ analyses the impact of the most recently obtain finest solution and $GBest_{i,j}$ is the most successful global solution to date. iter is the current iteration and $Max_{iter}$ is how many iterations can be carried out. ε is a small value that denotes the level of food quality available, $CPD_{i,j}\;$is the combined impact of every PD in the colony, rand is a random number between 0 and 1, and PE is the predator effect [35].

(30) $$PE=1.5\times {\left(1-\frac{iter}{Max_{iter}}\right)}^{\left(2\frac{iter}{Max_{iter}}\right)}$$

##### PDO Pseudo-Code

**Figure 4:**
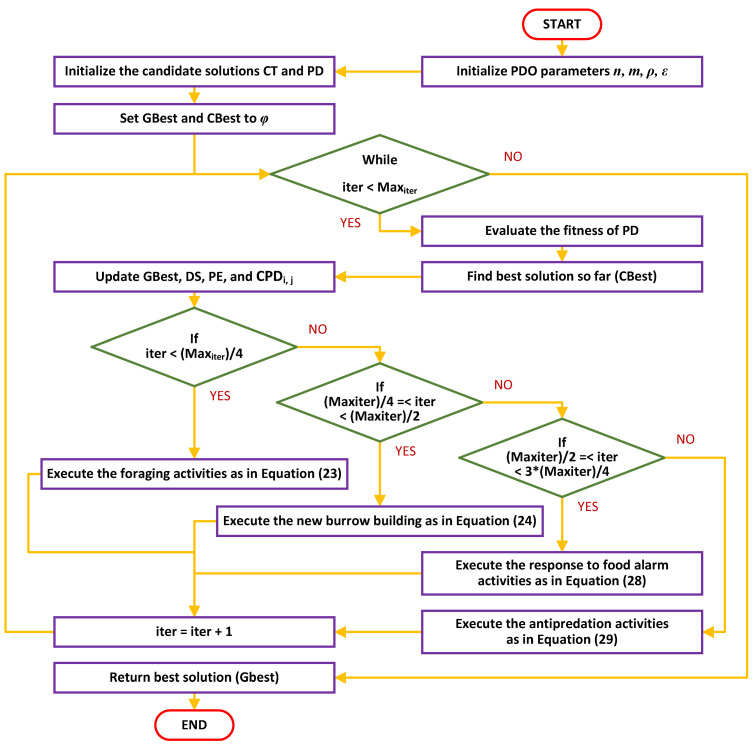
Block diagram representation of the flowchart of the PDO algorithm.

In this section, the algorithm of the PDO technique (Algorithm 1) is presented with the help of pseudo-codes and a block diagram, as represented in Figure 4 [35].

**Algorithm 1:** Pseudo-Code of PDO**Initialization**           Set the PDO parameters: n, m, ρ, ε           Set GBest and CBest as ϕ           Initialize the candidate solutions CT and PD **While** $iter<Max_{iter}$ **do**           **For** (i=1 to m)           **For** (j=1 to n) **do**           Calculate the fitness of PD           Find the best solution so far (CBest)           Update GBest           Update DS and PE           Update $CPD_{i,j}$,           **If** ($iter<\frac{Max_{iter}}{4}$) **then {foraging activities}**                    $PD_{i+1,j+1}=GBest_{i,j}-eCBest_{i,j}*\rho -CPD_{i,j}*Levy\left(n\right)$           **Else if** $\left(\frac{Max_{iter}}{4}\leq iter<\frac{Max_{iter}}{2}\right)$ **then {burrowing activities}**                            $PD_{i+1,j+1}=GBest_{i,j}*eCBest_{i,j}*DS*Levy\left(n\right)$           **Else if** $\left(\frac{Max_{iter}}{2}\leq iter<3\frac{Max_{iter}}{4}\right)$ **then {food alarm}**                        $PD_{i+1,j+1}=GBest_{i,j}-eCBest_{i,j}*\varepsilon -CPD_{i,j}*rand$           **Else {antipredation alarm}**                                   $PD_{i+1,j+1}=GBest_{i,j}*PE*rand$           **End If**           **End For**           **End For** iter=iter+1 **End While** **Return** the best solution $\left(GBest\right)$ **End**

#### 3.4.3. Implementation of PDO Algorithm for Optimal Tuning of the PI Gain Parameters

In this study, the PDO method has been applied to accomplish the optimal operation of HRES comprising PV, FC, and batteries. The gain values of the optimum PI parameters are determined with the aid of the PDO technique. The three objective functions given in Equations (31)–(33) have been minimized using the integral time absolute error (ITAE) standard.

**Figure 5:**
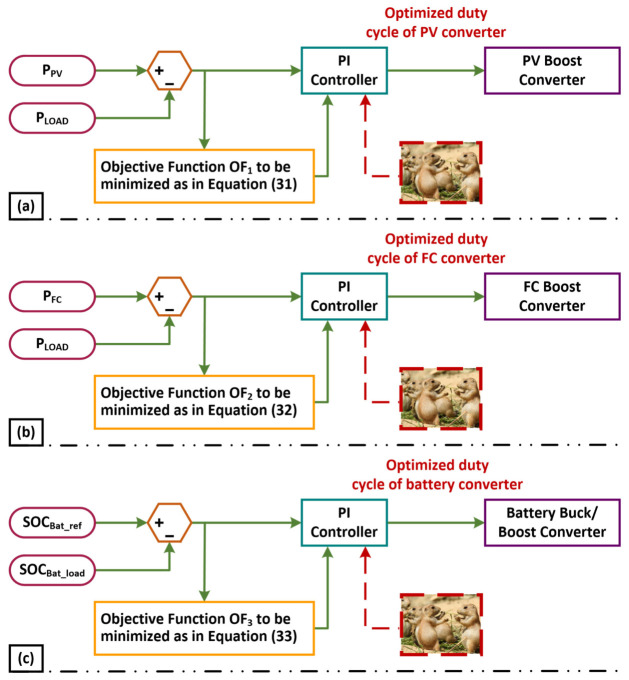
Mechanism of duty cycle generation for inverters with proposed PDO method: (**a**) PV, (**b**) FC, and (**c**) battery.

Figure 5a–c illustrates the mechanism of the generation of the duty cycle for the inverters of the PV, FC, and battery, respectively, using the suggested MWWO approach. The objective functions for the PV ($OF_{1}$), FC ($OF_{2}$), and battery ($OF_{3}$) are given below:

(31) $$OF_{1}=\int_{0}^{t}{\left({\mathrm{Er}}_{1}\left(t\right)\right)}^{2}\mathrm{dt}$$

(32) $$OF_{2}=\int_{0}^{t}{\left({\mathrm{Er}}_{2}\left(t\right)\right)}^{2}\mathrm{dt}$$

(33) $$OF_{3}=\int_{0}^{t}{\left({\mathrm{Er}}_{2}\left(t\right)\right)}^{2}\mathrm{dt}$$

where

(34) $${\mathrm{Er}}_{1}\left(t\right)\;=\Delta P=P_{PV}-P_{LOAD}$$

(35) $${\mathrm{Er}}_{2}\left(t\right)\;=\Delta P=P_{FC}-P_{LOAD}$$

(36) $${\mathrm{Er}}_{2}\left(t\right)\;=\Delta \;SOC_{bat}\;=SOC_{bat\_ref}-SOC_{bat\_load}$$

## 4. Matlab/Simulink Model and Results Discussion

### 4.1. Simulink Model and Description

**Figure 6:**
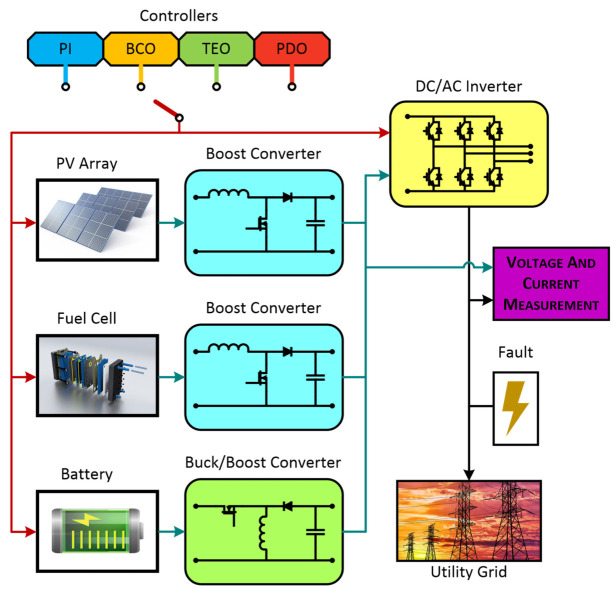
Simulink model of HRES-based MG.

In this research article, an HRES system comprising PV-, FC-, and battery-based distributed generations forming an MG system has been taken into consideration. PEIs such as DC-DC boost converters (for PV/FC), DC-DC buck/boost converters (for battery), and DCAC converters have been implemented. All the system components forming the MG system have been designed using MATLAB/Simulink architecture, and the values of each component are given in the appendix. The simulations have been carried out and various system responses have been observed for the traditional as well as proposed controller methods. In this research article, a simplified modelling approach has been adopted to address the specific challenges of improving control, achieving better transient response, and enhancing the power quality of a hybrid MG. Figure 6 below illustrates the diagrammatic representation of the system considered for study in this research paper.

### 4.2. Simulation Results and Discussion

The performance of the HRES-based MG has been studied and the results have been critically analysed by categorizing all the simulation results obtained into (1) HRES performance at the supply side and (2) system performance under fault scenarios at the grid side.

#### 4.2.1. HRES Performance on Supply Side

**Figure 7:**
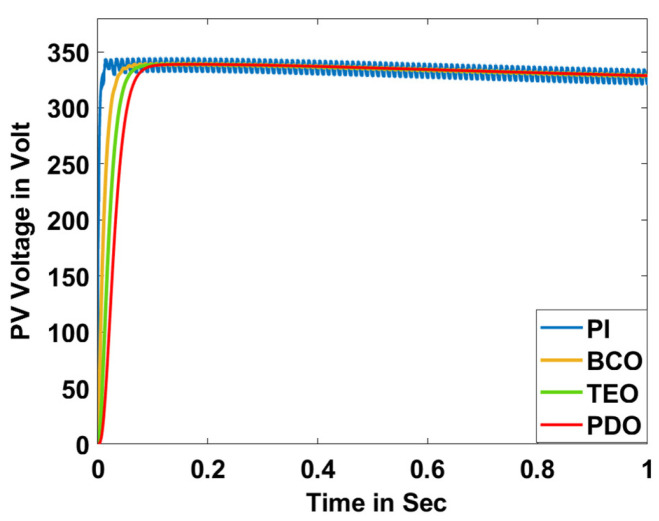
PV voltage.

**Figure 8:**
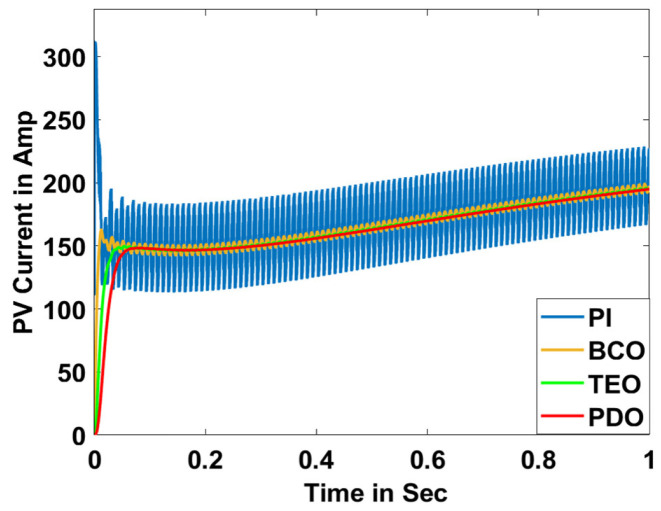
PV current.

**Figure 9:**
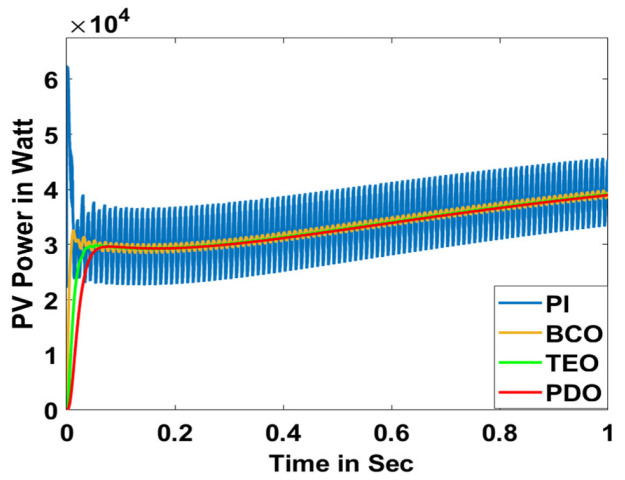
PV power.

**Figure 10:**
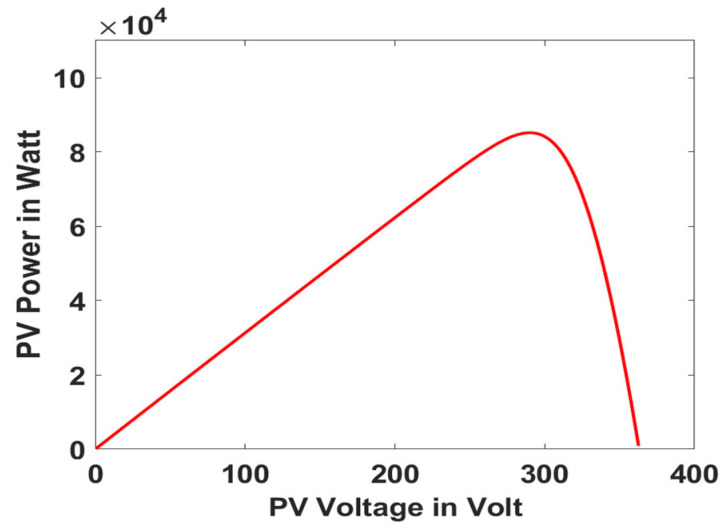
PV power vs. voltage.

**Figure 11:**
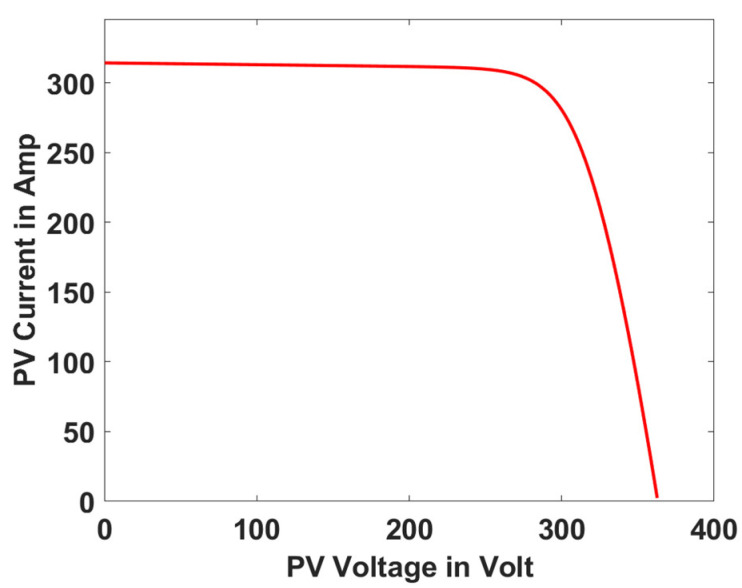
PV current vs. voltage.

**Figure 12:**
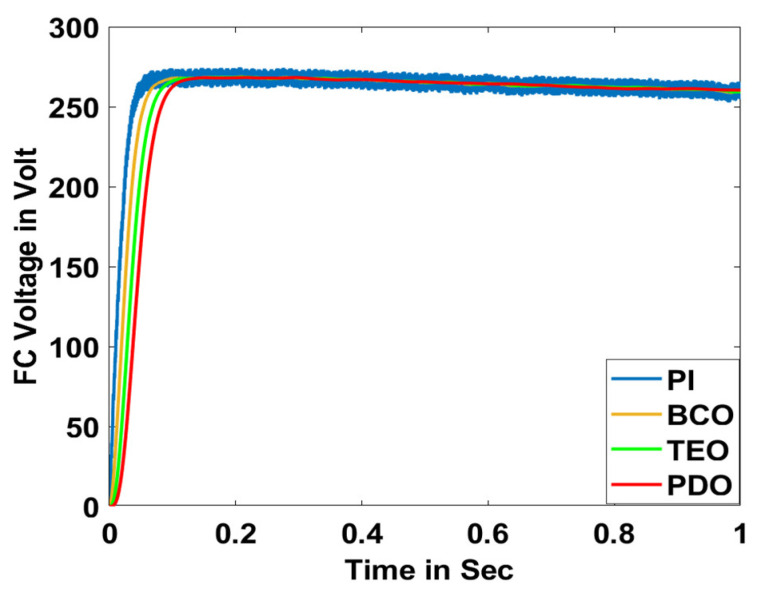
FC voltage.

**Figure 13:**
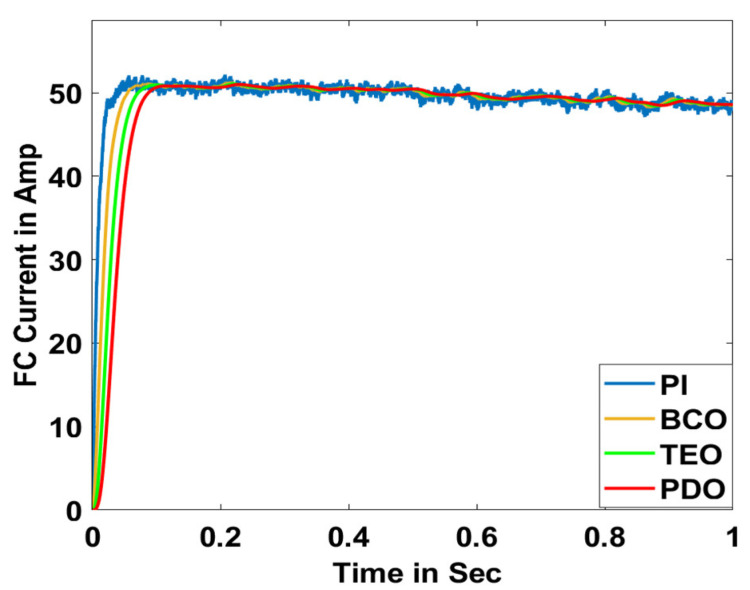
FC current.

**Figure 14:**
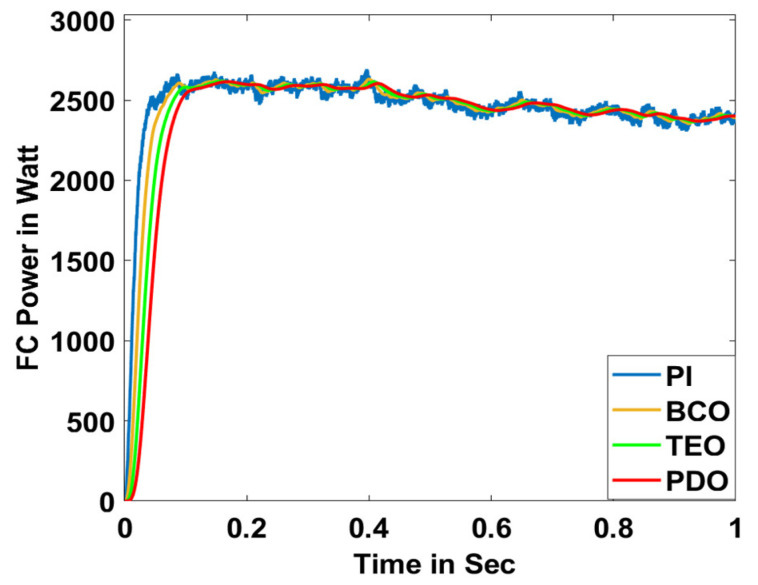
FC power.

**Figure 15:**
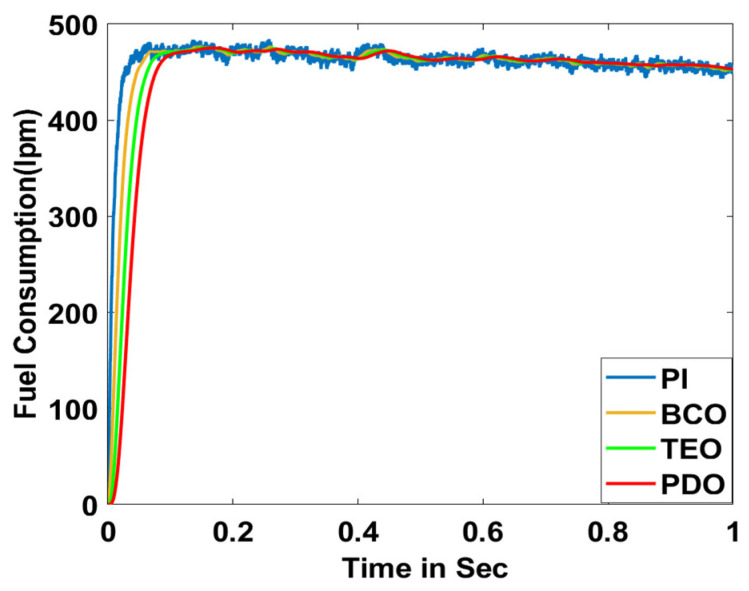
FC fuel consumption.

**Figure 16:**
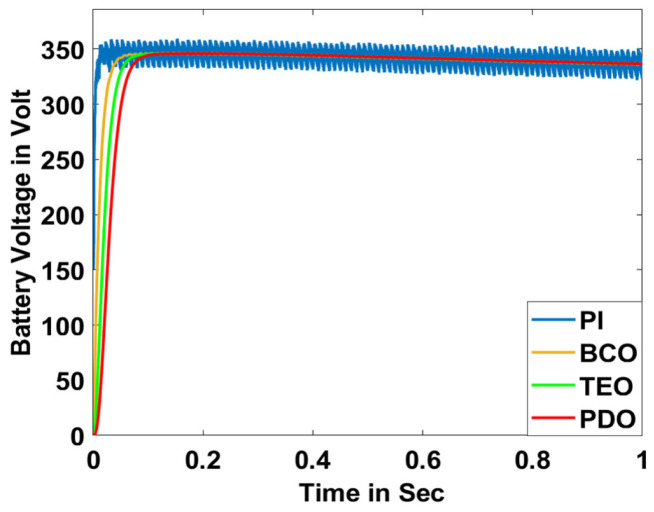
Battery voltage.

**Figure 17:**
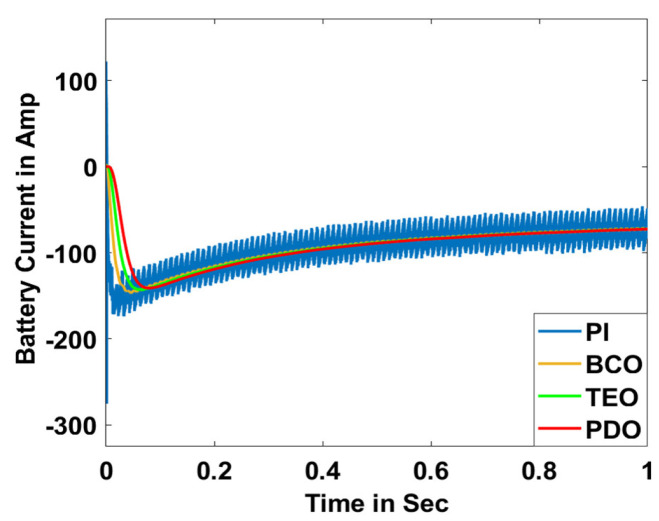
Battery current.

**Figure 18:**
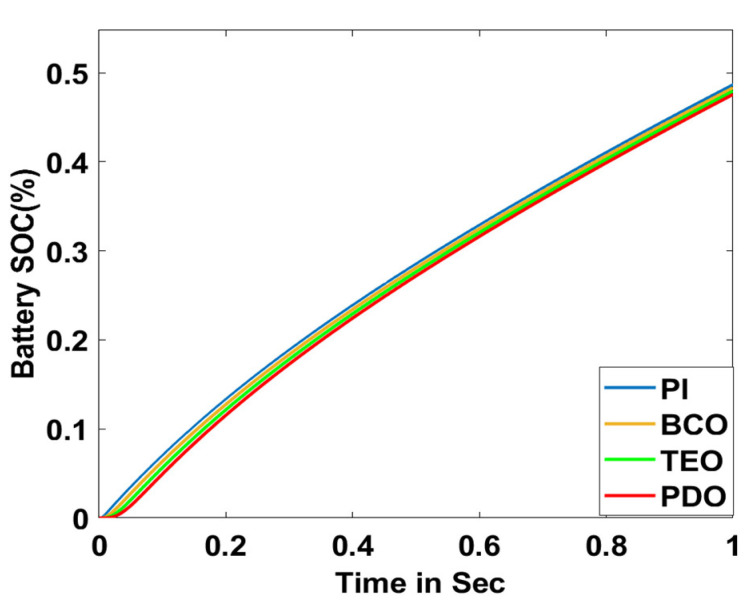
Battery SOC.

In this section, the performance and characteristics of the HRESs consisting of PV, FC, and battery (supply end) are presented, considering the proposed PDO controller as well as the conventional TEO, BCO, and PI methods. Figure 7, Figure 8 and Figure 9 depict the simulation results of the voltage, current, and power of the PV system, highlighting the comparison between the suggested and the traditional methods. The results indicate that the PV system response for the proposed PDO technique is better than the conventional TEO, BCO, and PI techniques in terms of improved stability, fewer harmonics, and enhanced system response. Figure 10 and Figure 11 indicate the power vs. voltage and current vs. voltage curves of the PV system for the suggested PDO controller. The FC system characteristics such as FC voltage, FC current, FC power, and FC fuel consumption are illustrated in Figure 12, Figure 13, Figure 14 and Figure 15, respectively. It can be inferred from the figures that the PDO-optimized PI outperforms the classical TEO, BCO, and PI methods in terms of reduced oscillations, better system reliability, and less fuel consumption. Battery voltage, current, and state of charge (SOC) responses are shown in Figure 16, Figure 17 and Figure 18, respectively. It can be concluded from the simulation results that the PDO algorithm robustly tunes the gain parameters of the PI controller, ensuring enhanced system overall dynamics, better efficacy, and reduced harmonics as compared to the TEO, BCO, and PI controllers.

#### 4.2.2. HRES Performance under Fault Scenarios at Grid Side

In this section, the system performance and characteristics of the HRES-based MG unit have been comprehensively studied in the grid-tied mode of operation. To validate the effectiveness, robustness, and efficacy of the proposed PDO algorithm compared to the conventional TEO, BCO, and PI methods, the MG system has been tested on four types of severe intentional PQ issues: (1) swell, (2) unbalanced, (3) oscillatory, and (4) notch conditions. A thorough description of the method of formation of these four PQ disturbances is also presented. Furthermore, simulation results for all four scenarios have been determined, and different system dynamics such as DC-link voltage, terminal voltage, voltage deviation, frequency, power factor, THD, grid active power, grid reactive power, grid apparent power, grid voltage and grid current are presented with regard to the suggested and classical control methods. Comprehensive study and investigation of the simulation results indicate that the PI controller tuned through the proposed PDO method surpasses the conventional TEO, BCO, and PI techniques in remarkably boosting the overall system efficacy and reliability, enhancing the PQ by reducing the harmonics, maintaining robust control of the active, reactive, and apparent power, improving the power factor, minimizing the voltage deviation, and keeping the terminal voltage, DC-link voltage, grid voltage, and grid current constant even with the occurrence of severe PQ disturbances.

##### Scenario A: Swell Condition

**Figure 19:**
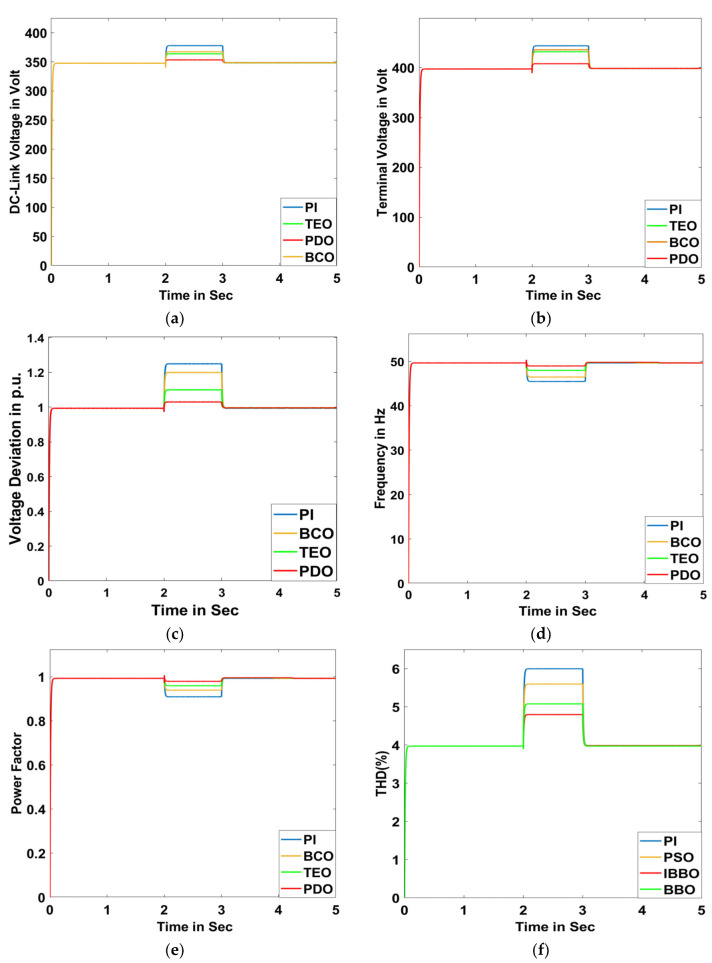

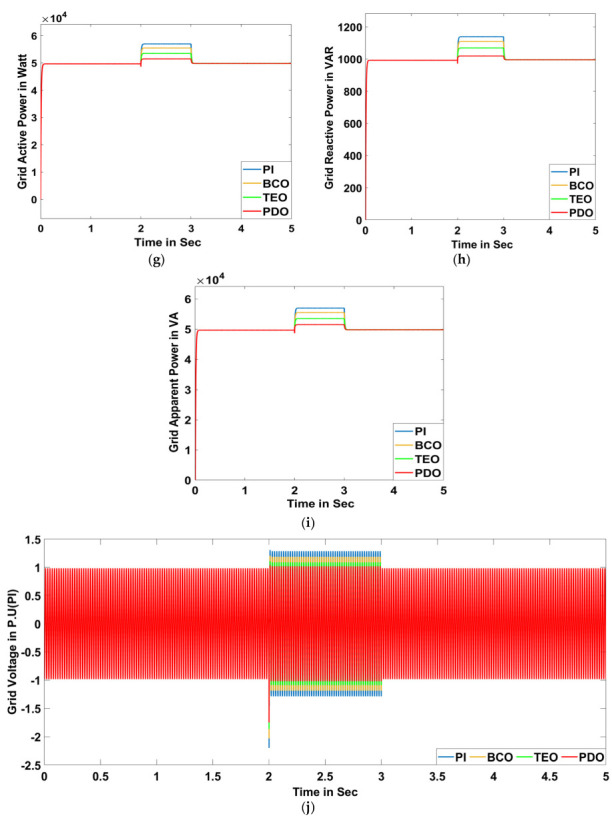

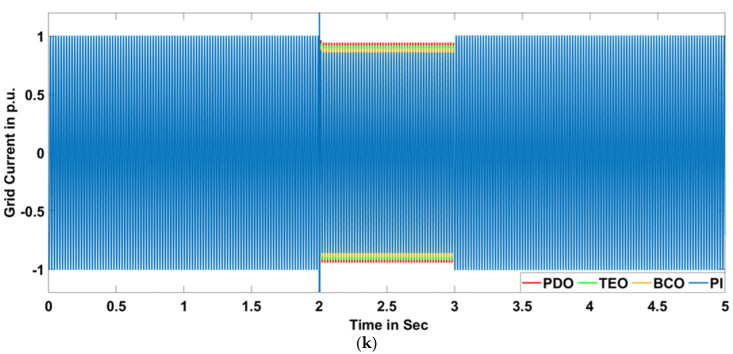
Swell Condition: (**a**) DC-link voltage, (**b**) terminal voltage, (**c**) voltage deviation, (**d**) frequency, (**e**) power factor, (**f**) THD, (**g**) grid active power, (**h**) grid reactive power, (**i**) grid apparent power, (**j**) grid voltage for PDO, TEO, BCO, and PI superimposed, and (**k**) grid current for PDO, TEO, BCO, and PI superimposed.

The swell phenomenon has been introduced through the removal of a heavy inductive load (2000 KVAR) for a time duration of 2 s to 3 s, and the model has been run for a time of 5 s. Different system characteristics such as DC-link voltage, terminal voltage, voltage deviation, frequency, power factor, THD, grid active power, grid reactive power, grid apparent power, grid voltage, and grid current are plotted in Figure 19a–k, respectively.

##### Scenario B: Unbalanced Condition

**Figure 20:**
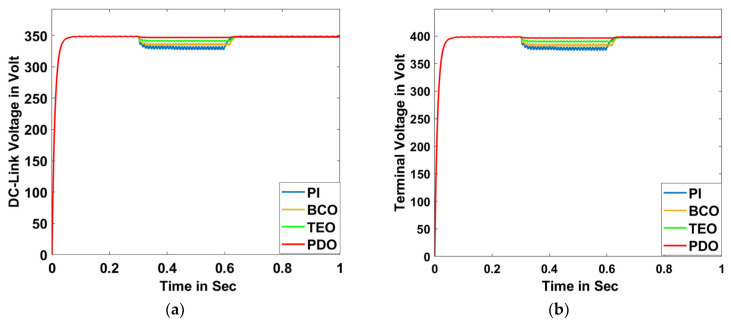

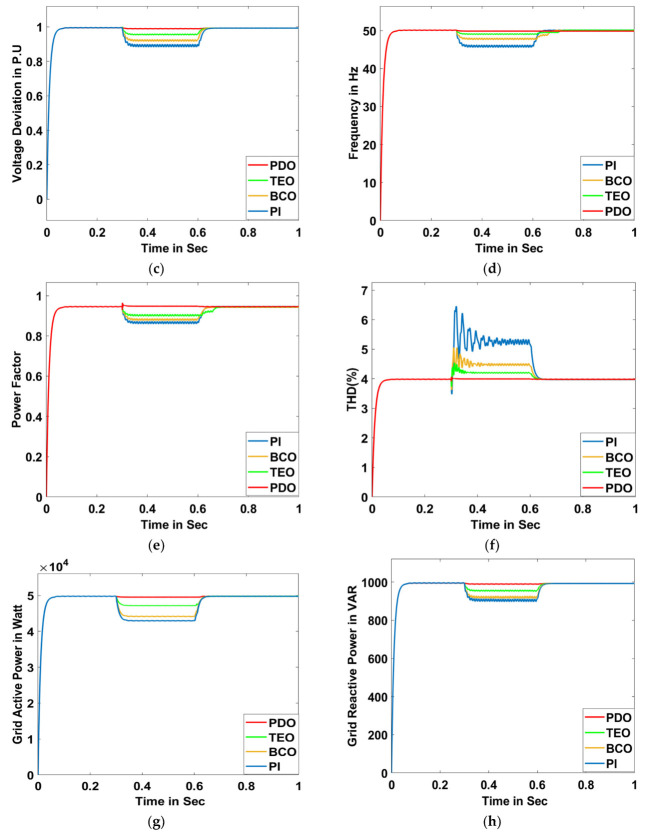

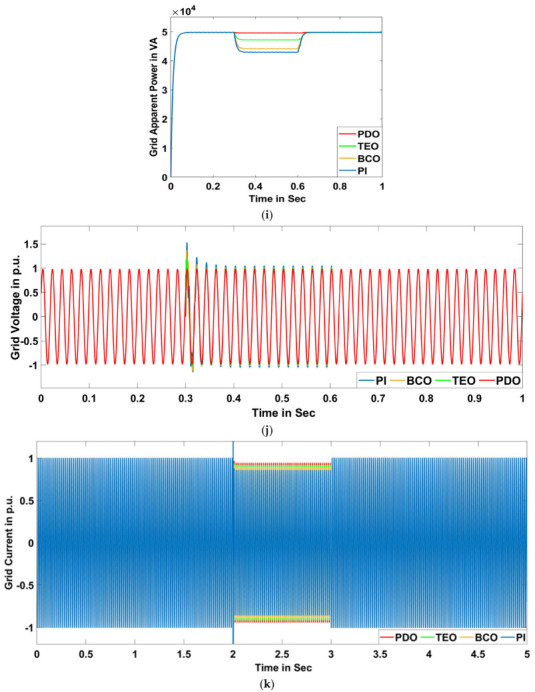
Unbalanced Condition: (**a**) DC-link voltage, (**b**) terminal voltage, (**c**) voltage deviation, (**d**) frequency, (**e**) power factor, (**f**) THD, (**g**) grid active power, (**h**) grid reactive power, (**i**) grid apparent power, (**j**) grid voltage for PDO, TEO, BCO, and PI superimposed, and (**k**) grid current for PDO, TEO, BCO, and PI superimposed.

The unbalanced voltage condition has been brought on by creating an unbalance in the voltage amplitude in one of the phases (phase-a) for a time of 0.3 s to 0.6 s, and the model has been run for 1 s. Various system responses such as DC-link voltage, terminal voltage, voltage deviation, frequency, power factor, THD, grid active power, grid reactive power, grid apparent power, grid voltage, and grid current are illustrated in Figure 20a–k, respectively.

##### Scenario C: Oscillatory Transient Condition

**Figure 21:**
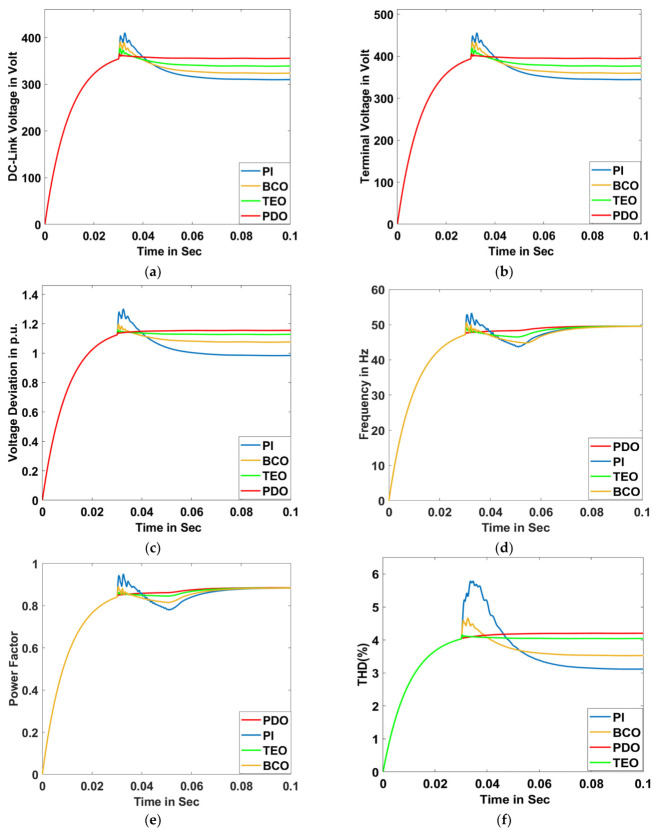

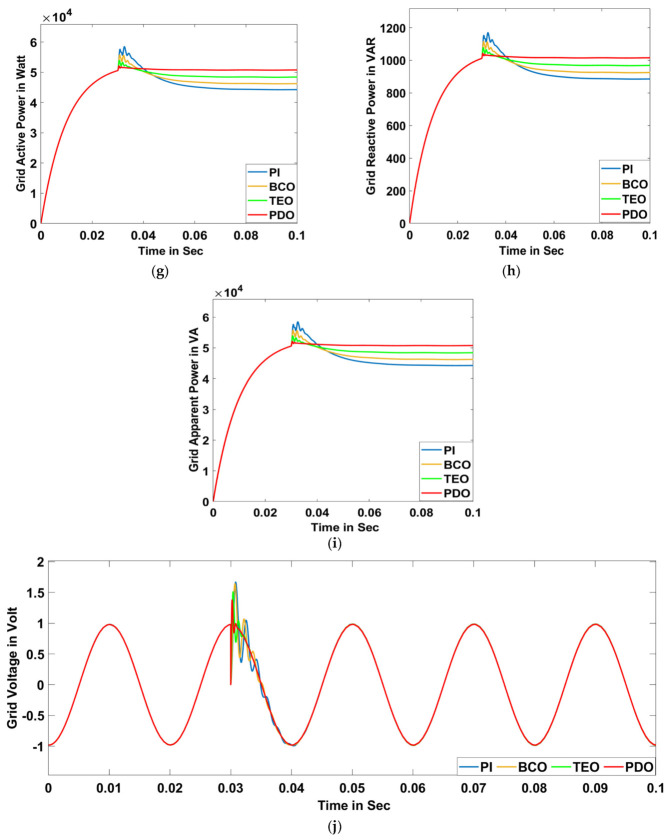

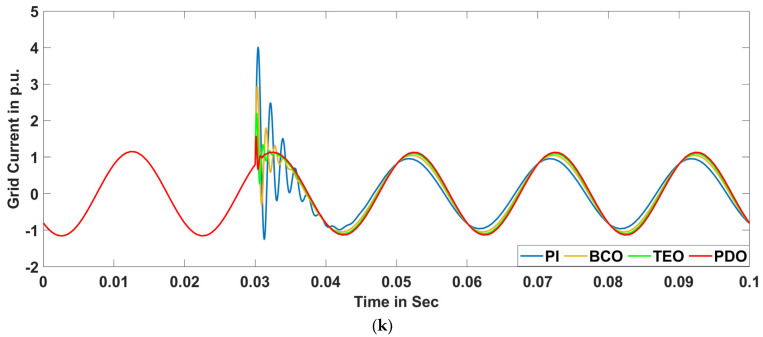
Oscillatory Transient Condition: (**a**) DC-link voltage, (**b**) terminal voltage, (**c**) voltage deviation, (**d**) frequency, (**e**) power factor, (**f**) THD, (**g**) grid active power, (**h**) grid reactive power, (**i**) grid apparent power, (**j**) grid voltage for PDO, TEO, BCO, and PI superimposed, and (**k**) grid current for PDO, TEO, BCO, and PI superimposed.

The oscillatory transient state has been created through the introduction of a capacitive load (750 KVAR) for a time duration of 0.03 s to 0.05 s, where the proposed model has been run for 0.1 s. Numerous system characteristics such as DC-link voltage, terminal voltage, voltage deviation, frequency, power factor, THD, grid active power, grid reactive power, grid apparent power, grid voltage, and grid current are plotted in Figure 21a–k, respectively.

##### Scenario D: Notch Condition

**Figure 22:**
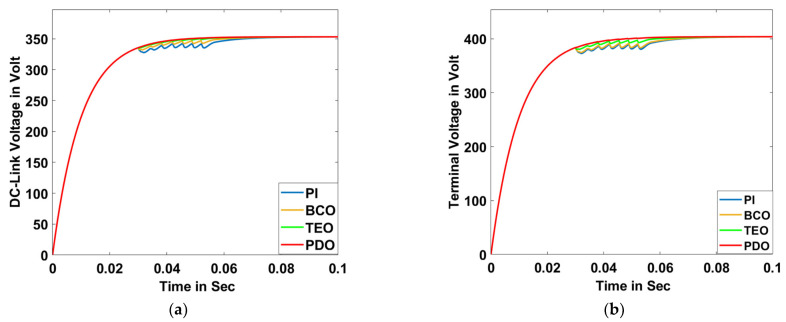

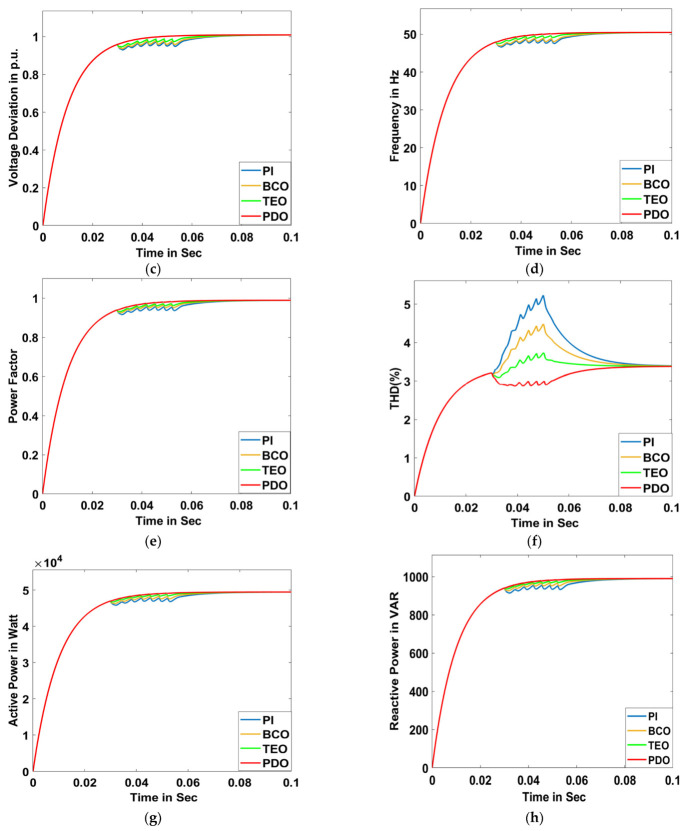

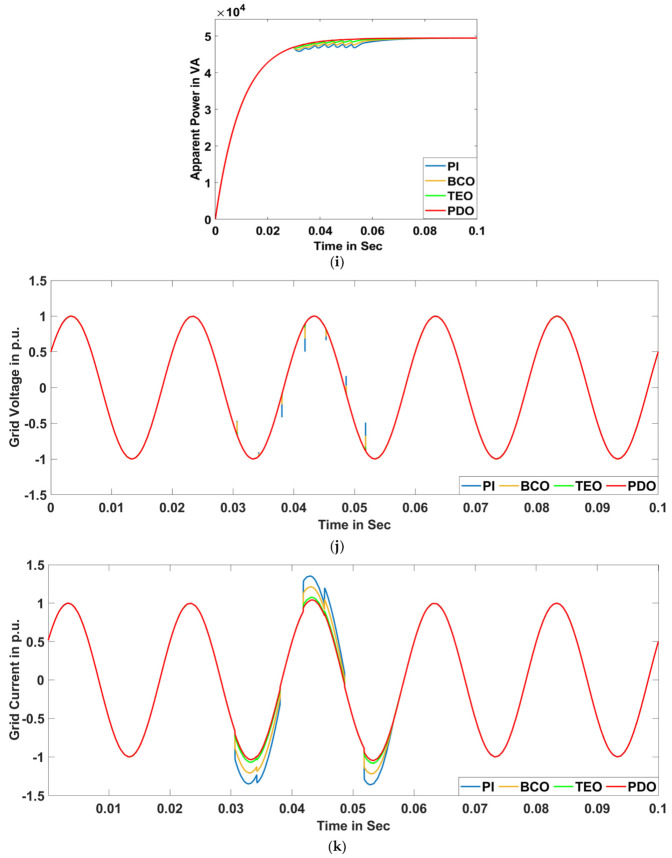
Notch Condition: (**a**) DC-link voltage, (**b**) terminal voltage, (**c**) voltage deviation, (**d**) frequency, (**e**) power factor, (**f**) THD, (**g**) grid active power, (**h**) grid reactive power, (**i**) grid apparent power, (**j**) grid voltage for PDO, TEO, BCO, and PI superimposed, and (**k**) grid current for PDO, TEO, BCO, and PI superimposed.

The notch phenomenon has been introduced with the help of a six-pulse three-phase rectifier for a duration of 0.03 s to 0.06 s while running the model for 0.1 s. Various system responses such as DC-link voltage, terminal voltage, voltage deviation, frequency, power factor, THD, grid active power, grid reactive power, grid apparent power, grid voltage, and grid current are illustrated in Figure 22a–k, respectively.

## 5. Conclusions and Future Scope

### 5.1. Conclusive Remarks

A growing field of research in grid-tied MG systems is PQ mitigation in HRESs comprising various renewable-based DGs (PV and FC) and energy storage systems (battery). The usage of PEIs, non-linear loads, unbalanced loads, and high-frequency switching properties on the load side in HRES power systems plays a significant role in affecting the quality of the power delivered and may result in the malfunctioning of the entire power system generation unit. To efficiently address the above issues, a robust and intelligent control scheme based on the prairie dog optimization algorithm has been implemented in this research article. The proposed PDO method facilitates optimal gain scheduling of the PI controllers, which are utilized to drive the inverter for governing the PCC voltage between the MG and the DGs, which, in turn, effectively mitigates the PQ issues.

The MG system has been designed in the MATLAB/Simulink environment, and various intentional fault cases such as swell, unbalanced load, oscillatory transient, and notch have been studied to prove the efficacy of the proposed controller in maintaining the dynamic voltage stabilization, mitigating the harmonics distortion, and reducing the consumed reactive power. Various system characteristics such as terminal voltage, DC-link voltage, voltage deviation, active power, reactive power, apparent power, THD, power factor, frequency, grid voltage, and grid current have been plotted. The system responses of the proposed PDO method have been compared with two other conventional OTs: the BCO and TEO techniques. A thorough examination of the findings supports the effectiveness, reliability, and ability of the proposed PDO controller in terms of reducing THD, improving active and reactive power control, boosting power factor, reducing voltage deviation, and keeping terminal voltage, DC-link voltage, grid voltage, and grid current almost constant in the incident of PQ faults, thus paving the way for its real-time deployment in an MG system.

### 5.2. Comparative Numerical Value Analysis Justifying Efficacy of Proposed Method

In this section, a critical analysis of the characteristics obtained from the simulation has been carried out, and the numerical values obtained after a thorough study are presented in a tabulated manner to support the claim of the robustness of the proposed PDO method over other traditional methods. The four types of PQ issues, (1) swell, (2) unbalanced load, (3) oscillatory transient, and (4) notch, have been introduced for justification of the robustness of the proposed PDO controller. The numerical value response for various system dynamics such as (a) DC-link voltage, (b) terminal voltage, (c) voltage deviation, (d) frequency, (e) power factor, (f) THD, (g) grid active power, (h) grid reactive power, (i) grid apparent power, (j) grid voltage, and (k) grid current is presented in tabular form for the swell, unbalanced, oscillatory transient, and notch conditions in Table 2, Table 3, Table 4 and Table 5, respectively. A comprehensive study of the numerical values obtained justifies the robustness of the suggested PDO controller in terms of optimally tuned the PI gain parameters, minimum voltage deviation, enhanced reactive power, reduced THD, improved power factor, and almost constant frequency, even when subjected to severe PQ issues.

### 5.3. Future Directions

The following are some probable upcoming research initiatives that could be investigated:

1. Only single-objective continuous optimization issues have been solved by the proposed PDO, and scientists may consider creating the binary version of the technique.
2. PDO’s multi-objective form may also be established.
3. Researchers may also explore the idea of enhancing and hybridizing PDO with other available techniques.
4. PDO can be expanded to address additional discrete or continuous real-world issues, which is a potential effort for researchers to embark on.
5. Several distributed generating technologies, such as wind, biomass, microturbines, etc., can be integrated with PV and FC to expand the capacity of supplying power.
6. Other energy storage units such as supercapacitors, flywheels, compressed air, etc. can be utilized and their response can be studied.

## Figures and Tables

**Table 1:** Summary of existing popular contemporary algorithms highlighting merits and demerits.

Control Strategy/Algorithm	Merits	Demerits
Proportional Integral	Implementation is straightforward. The system exhibits a linear response. No steady-state error is present.	Fails to effectively handle nonlinear and imbalanced systems. The range of stability is extremely limited. The dynamic and transient response is unsatisfactory.
Harris Hawks Algorithm	Effective exploration. Possesses a good balance between exploitation and exploration. Exhibits rapid convergence rates.	Used for a specified application domain. Usually influenced by the choice of parameter settings. In certain scenarios, it has a relatively limited theoretical foundation. Lacks thorough benchmarking on a variety of common optimization problems.
Grasshopper Optimization Algorithm	Performs robustly. Less sensitive to noise in the problem domain. Parallelization that enables effective use of the computer resources is possible.	Immune to the threat of converging local maxima. Performance is highly selective to a selection of parameters. Computationally expensive.
Genetic Algorithm	Effective in combinatorial optimization. The solutions provided are easily interpretable. Has multiplicity solution. Effective capability to handle constraints.	Susceptible to premature convergence. Longer computational time. Requires appropriate selection of parameters for efficient operation. Knowledge dependency to explore search space.
Particle Swarm Optimization	Capable of an effective search for global optima. Simple and easy to implement. Has a faster convergence rate. Robust in handling noisy or stochastic objective functions.	Prone to premature convergence. Sensitive to parameter settings. Difficult to handle constraints effectively. Computationally intensive especially for large-scale problems. Lack of diversity preservation as optimization progresses.
Atom Search Optimization	Global optimization capability. Simplicity. Fast convergence. Better adaptability.	Lack of extensive research and benchmarking. Complexity in the selection of its parameters. Sensitive to the selection of its parameters. Lack of theoretical foundation. Computational complexity.
Salp Swarm Optimization	Facilitates diverse exploration of the solution space. Inherent adaptability to dynamic environments. Ability to parallelize across multiple processors or computing nodes.	Low precision. Low optimization dimension. Slow convergence speed. Computationally expensive. Solutions are not easily interpretable by users.
Aquila Optimization	Better intensification and diversification capabilities. Fast convergence rate. Low residual errors. Strong scalabilities.	Local optima stagnation. Complex in implementation. Low convergence speed.
Fuzzy Logic Controller	Easy to calculate. Capable of accommodating system non-linearity. Capable of controlling a single or multiple input/output system.	Design is challenging. Relies on the expertise and experience of the practitioner. Requires appropriate parameter selection, the definition of membership functions, and fuzzy rules.
Adaptive Network Fuzzy Inference	Capable of adjusting parameters based on input–output data. Enhanced decision-making capabilities. Effective in handling nonlinearity in systems. Ability to provide transparent and interpretable models. Scalable and can handle large-scale and high-dimensional problems.	Complexity of model building. Requires a sizable amount of input–output training data for precise learning and generalization. Computationally demanding, particularly for complicated systems or large-scale problems with a lot of input variables. Face challenges in effectively addressing uncertainties in the input data or modelling process.
Green Leaf-hopper Flame Optimization	Adaptable with many sorts of constraints, problem structures, and objective functions. Relatively simple to understand and implement. Fast convergence rates, efficiently narrowing down the search space towards optimal solutions. Less sensitive to parameter settings and noise in the problem domain.	Due to its novelty, green leaf-hopper flame optimization has limited research and benchmarking across a wide range of problems compared to well-established algorithms. Lack of a well-established theoretical foundation. Computationally expensive for large-scale optimization problems.
Chaotic Butterfly Optimization	Ability to maintain a proper balance between exploitation and exploration. Robust performance in handling noisy or stochastic objective functions. Adaptability to dynamic problem domains.	Sensitive to the selection and tuning of its parameters. Cannot exploit problem-specific characteristics effectively. Being a new optimization method, in comparison to more established algorithms, its use and performance on a variety of benchmark issues have not been well examined.
Artificial Neural Network	Capable of efficiently simulating and capturing nonlinear interactions between the variables that affect the output. Ability to run several computations at once, allowing for information processing in parallel. Through the training process, it can adapt and learn from data. Capable of adapting and learning from data. Can provide insights into the internal workings and decision-making processes.	Lack of transparency in the decision-making process. Computationally expensive and time-consuming, especially for large and deep networks. Has hyperparameter sensitivity. Higher manufacturing costs. There can only be an input of numeric data. The effectiveness relies on the user’s skill and expertise.
Support Vector Machine	Effective in high-dimensional spaces. Robust to overfitting. Versatility in kernel functions. Effective with small training sets. Robust to outliers in the training data.	Sensitivity to parameter selection. Computationally intensive for large datasets. Lack of probabilistic output. Difficulty scaling with large feature spaces. Lack of incremental learning.
Model Predictive Control	Efficacy in handling constraints. Utilizes a predictive model of the system to make control decisions. Robustness to disturbances. Can effectively handle nonlinear systems. Effectiveness and wide applicability.	Sensitivity to model inaccuracies. Proper tuning of MPC controllers can be a challenging task. Requirement of accurate measurements. Sensitivity to time delays. Implementation/maintenance effort is needed.
Multi-Agent System	Allows distributed problem solving. Exhibits flexibility and adaptability to changing environments and requirements. Ability to scale to large systems with numerous agents. Enables dynamic task allocation and load balancing between agents. Allows heterogeneity and promotes specialization.	Complexity in design and implementation. Relies on agent communication for information sharing, coordination, and collaboration. Lack of global knowledge. Sensitivity to agent heterogeneity. Multiple autonomous agents that are involved can introduce security and privacy risks. The computational requirements of MAS can be significant, especially in systems with numerous agents and complex decision-making processes.
Amended Penguin Optimization	Demonstrates rapid convergence. Good balance between exploration and exploitation. Can handle a wide range of optimization problems. Implementation does not require complex mathematical formulations and is relatively straightforward.	Relatively limited research attention and application. Computational complexity may increase with the size and complexity of the optimization problem. May occasionally converge to local optima. Limited support for constraints. Sensitivity to initial population. Performance and robustness have not been extensively validated across a wide range of benchmark problems and real-world applications.
Squirrel Search Algorithm	Excels in exploration capabilities. Maintains population diversity, preventing premature convergence to suboptimal solutions. Robustness in handling noisy or uncertain problem landscapes. Exhibits fast convergence properties. Has a small number of control parameters, making it relatively easy to implement and tune.	Sensitivity to parameter settings. Lack of extensive validation. Convergence to local optima. Does not have inherent problem-specific adaptation mechanisms. Lack of scalability studies. Balancing exploration and exploitation is a common challenge.

**Table 2:** Comparative numerical value analysis of different system responses for proposed PDO controller with TEO, BCO, and PI controller methods for swell condition.

Type of Controller → System Values ↓	PI		BCO		TEO		Proposed PDO	
PI Controller Gains	*K_p_*	*K_i_*	*K_p_*	*K_i_*	*K_p_*	*K_i_*	*K_p_*	*K_i_*
	0.087	0.0032	0.167	0.0061	0.461	0.0054	0.557	0.0089
Terminal Voltage (Volt)	445		436		431		408	
DC-Link Voltage (Volt)	378		367		364		353	
Voltage Deviation (p.u.)	1.25		1.2		1.1		1.03	
Active Power (Watt)	57,000		55,500		53,450		48,200	
Reactive Power (Var)	890		921		948		977	
Apparent Power (VA)	57,150		55,640		53,300		48,050	
THD (%)	6.09		5.83		5.15		4.67	
Power Factor	0.91		0.94		0.965		0.98	
Frequency (Hz)	45.47		46.45		48.2		49.1	
Grid Voltage (p.u.)	1.287		1.19		1.089		1.02	
Grid Current	0.862		0.895		0.92		0.945	

**Table 3:** Comparative numerical value analysis of different system responses for proposed PDO controller with TEO, BCO, and PI controller methods for unbalanced condition.

Type of Controller → System Values ↓	PI		BCO		TEO		Proposed PDO	
PI Controller Gains	*K_p_*	*K_i_*	*K_p_*	*K_i_*	*K_p_*	*K_i_*	*K_p_*	*K_i_*
	0.087	0.0032	0.134	0.0087	0.249	0.0071	0.432	0.0046
Terminal Voltage (Volt)	378		385		392		397	
DC-Link Voltage (Volt)	332		338		343		348	
Voltage Deviation (p.u.)	0.90		0.93		0.97		0.99	
Active Power (Watt)	43,700		44,550		47,450		49,500	
Reactive Power (Var)	941		966		975		993	
Apparent Power (VA)	43,510		44,530		47,280		49,450	
THD (%)	6.23		5.14		4.56		4.02	
Power Factor	0.87		0.89		0.91		0.95	
Frequency (Hz)	46.71		48.23		49.13		49.59	
Grid Voltage (p.u.)	1.48		1.39		1.18		1.01	
Grid Current	2.85		2.23		1.57		1.01	

**Table 4:** Comparative numerical value analysis of different system responses for proposed PDO controller with TEO, BCO, and PI controller methods for oscillatory transient condition.

Type of Controller → System Values ↓	PI		BCO		TEO		Proposed PDO	
PI Controller Gains	*K_p_*	*K_i_*	*K_p_*	*K_i_*	*K_p_*	*K_i_*	*K_p_*	*K_i_*
	0.087	0.0032	0.149	0.0093	0.483	0.0089	0.798	0.0091
Terminal Voltage (Volt)	361		373		381		400	
DC-Link Voltage (Volt)	317		324		335		350	
Voltage Deviation (p.u.)	1.16		1.14		1.09		1.01	
Active Power (Watt)	47,910		49,630		50,560		51,100	
Reactive Power (Var)	934		956		969		984	
Apparent Power (VA)	48,040		49,806		50,720		51,340	
THD (%)	5.98		4.51		4.12		4.05	
Power Factor	0.795		0.819		0.83		0.89	
Frequency (Hz)	49.01		49.25		49.66		49.96	
Grid Voltage (p.u.)	1.62		1.51		1.19		1.01	
Grid Current	4.0		3.1		1.61		1.05	

**Table 5:** Comparative numerical value analysis of different system responses for proposed PDO controller with TEO, BCO, and PI controller methods for notch condition.

Type of Controllers → System Values ↓	PI		BCO		TEO		Proposed PDO	
PI Controller Gains	*K_p_*	*K_i_*	*K_p_*	*K_i_*	*K_p_*	*K_i_*	*K_p_*	*K_i_*
	0.087	0.0032	0.129	0.0098	0.321	0.0082	0.673	0.0067
Terminal Voltage (Volt)	378		386		392		398	
DC-Link Voltage (Volt)	336		341		345		350	
Voltage Deviation (p.u.)	1.06		1.04		1.02		1.001	
Active Power (Watt)	46,700		47,030		48,100		49,100	
Reactive Power (Var)	948		962		976		990	
Apparent Power (VA)	46,110		46,820		48,280		49,920	
THD (%)	5.15		4.23		3.48		2.96	
Power Factor	0.94		0.955		0.961		0.987	
Frequency (Hz)	51.57		51.05		50.83		50.16	
Grid Voltage (p.u.)	0.58		0.73		0.86		1.02	
Grid Current	1.35		1.215		1.05		1.01	

## Data Availability

Authors may provide data upon request.

## Abbreviations

**List of Acronyms**			
HRES	Hybrid Renewable Energy Source	PEIs	Power Electronics Interfaces
MG	Microgrid	PID	Proportional Integral Derivative
PQ	Power Quality	DVR	Dynamic Voltage Regulator
PDO	Prairie Dog Optimization	DS	Digging Strength
PV	Photovoltaic	PEM	Proton Exchange Membrane
FC	Fuel Cell	KVL	Kirchhoff’s Voltage Law
PI	Proportional Integral	PWM	Pulse Width Modulation
BCO	Bee Colony Optimization	PDs	Prairie Dogs
TEO	Thermal Exchange Optimization	CT	Coterie
RESs	Renewable Energy Sources	ITAE	Integral Time Absolute Error
CHP	Combined heat and power	OFs	Objective Functions
AC	Alternating Current	SOC	State of Charge
DC	Direct Current	THD	Total Harmonic Distortion
**List of Symbols**			
I	terminal current of the PV module		
$I_{L}$	current generated by the PV cell (photocurrent)		
$I_{0}$	diode saturation current		
q	charge of an electron		
$V_{D}$	diode voltage		
n	number of PV cells		
K	Boltzmann constant		
T	actual temperature in Kelvin		
$V_{\;}$	output voltage of PV		
$R_{sh}$	shunt resistance		
U	output voltage of FC		
$V_{1}$	activation overvoltage of FC		
$V_{2}$	concentration overvoltage of FC		
$V_{3}$	voltage drop across output resistor of FC		
N	number of stacked cells of FC		
R	universal gas constant (JK/kmol)		
F	Faraday constant		
$PH_{2}$	mole fraction of the species of hydrogen		
$PO_{2}$	mole fraction of the species of oxygen		
$PH_{2}O$	mole fraction of the species of water		
$V_{T}\left(t\right)$	terminal voltage of battery		
$V_{OC}\left(z\left(t\right)\right)$	internal open-circuit voltage source of battery		
$R_{S}$	internal resistance of battery		
$I\left(t\right)$	current through battery		
$D_{T}$	duty cycle		
$K_{p}$	proportional gain of PI controller		
$K_{i}$	integral gain of PI controller		
$e\left(t\right)$	error function of PI controller		
$s_{n}$	normalized value of a partial or full solution		
$s_{mn}$	highest value of the overall partial/complete solution		
i	number of forward passes		
$s_{a}$	promoted solution		
R	number of bees that are recruiters		
h	heat transfer coefficient		
A	heat flow area		
$T_{0}$	high temperature		
$T_{b}$	constant temperature		
h	temperature coefficient		
Q	heat loss from the surface		
ρ	density		
c	specific heat		
m	number of coteries		
n	number of prairie dogs		
d	dimensional space by a vector		
$CT_{i,j}$	ith coterie’s jth dimension		
$PD_{i,j}$	ith prairie dog in a coterie’s jth dimension		
$UB_{j}$	r introduces the stochastic property to confirm exploration		
$LB_{j}$	lower bounds of the jth dimension		
$f\left(PD\right)$	fitness function value for each PD’s location		
$eCBest_{i,j}$	evaluates the effects of the most effective solution currently acquired worldwide		
$GBest_{i,j}$	best solution currently available globally		
rPD	location of a random solution		
$CPD_{i,j}$	randomized cumulative effect of all prairie dogs in the colony		
ρ	experiment’s specialized food source alarm		
DS	coterie’s digging strength		
Δ	modest number that signifies disparities that occur among the PDs		
r	introduces the stochastic property to confirm exploration		
iter	current iteration		
$Max_{iter}$	maximum number of iterations		
ε	small value that denotes the level of food quality available		
rand	random number		
PE	predator effect		

## Appendix A

**Table:**

PV Panel	PEMFC	Boost Converter	Battery	Utility Grid	PDO Algorithm
Max. power: 164.85 watt, parallel strings: 50, cells per module: 72, OC voltage: 43.5 volt, SC current: 5.25 amp, light-generated current: 5.2764 A, shunt resistance: 125.0069.05 ohm, series resistance: 0.62818 ohm	Nominal stack power: 60,000 watt, resistance of fuel cell: 0.46801 ohm, Nernst voltage of cell: 1.1243 volt, system temperature: 321 kelvin, air supply pressure: 1 bar	Inductance: 254 mH, capacitance: 42 µF	Nominal voltage: 600 volt, rated capacity: 80,000 Ah, initial state of charge: 40%, battery response time: 0.03 s	Phase to phase voltage (RMS): 400 volt, frequency: 50 Hz, phase angle of phase-a: 0 degree	Number of coteries (m): 30, number of prairie dogs: 100, maximum number of iterations: 1000, specialized food source alarm (*ρ*): 0.1 KHz, stochastic property (*r*): (−1 or +1), random number (*rand*): (0 to 1)
